# An ATP-responsive metabolic cassette comprised of inositol tris/tetrakisphosphate kinase 1 (ITPK1) and inositol pentakisphosphate 2-kinase (IPK1) buffers diphosphosphoinositol phosphate levels

**DOI:** 10.1042/BCJ20200423

**Published:** 2020-07-24

**Authors:** Hayley Whitfield, Gaye White, Colleen Sprigg, Andrew M. Riley, Barry V.L. Potter, Andrew M. Hemmings, Charles A. Brearley

**Affiliations:** 1 School of Biological Sciences, UEA, Norwich Research Park, Norwich NR4 7TJ, U.K.; 2 Medicinal Chemistry and Drug Discovery, Department of Pharmacology, University of Oxford, Mansfield Road, Oxford OX1 3QT, U.K.; 3 School of Chemistry, UEA, Norwich Research Park, Norwich NR4 7TJ, U.K.

**Keywords:** diphosphoinositol phosphates, energy charge, IPK1, ITPK1, phosphate homeostasis

## Abstract

Inositol polyphosphates are ubiquitous molecular signals in metazoans, as are their pyrophosphorylated derivatives that bear a so-called ‘high-energy’ phosphoanhydride bond. A structural rationale is provided for the ability of Arabidopsis inositol tris/tetrakisphosphate kinase 1 to discriminate between symmetric and enantiomeric substrates in the production of diverse symmetric and asymmetric *myo*-inositol phosphate and diphospho-*myo*-inositol phosphate (inositol pyrophosphate) products. Simple tools are applied to chromatographic resolution and detection of known and novel diphosphoinositol phosphates without resort to radiolabeling approaches. It is shown that inositol tris/tetrakisphosphate kinase 1 and inositol pentakisphosphate 2-kinase comprise a reversible metabolic cassette converting Ins(3,4,5,6)P_4_ into 5-InsP_7_ and back in a nucleotide-dependent manner. Thus, inositol tris/tetrakisphosphate kinase 1 is a nexus of bioenergetics status and inositol polyphosphate/diphosphoinositol phosphate metabolism. As such, it commands a role in plants that evolution has assigned to a different class of enzyme in mammalian cells. The findings and the methods described will enable a full appraisal of the role of diphosphoinositol phosphates in plants and particularly the relative contribution of reversible inositol phosphate hydroxykinase and inositol phosphate phosphokinase activities to plant physiology.

## Introduction

*Myo*-inositol hexakisphosphate (InsP_6_) is the predominant form of phosphorus storage molecule in plants, where in storage organs and tissues it may accumulate to several percent of dry weight [1]. The enzymology of InsP_6_ synthesis extends to families of enzymes collectively capable of phosphorylating all six hydroxyls of the inositol ring [2]. These include inositol kinase [3] and assorted inositol phosphate (hydroxy) kinases, including inositol 1,3,4-trisphosphate 5/6-kinases, also known as inositol tris/tetrakisphosphate kinases, ITPKs [4–6]; inositol polyphosphate kinases [7], commonly called IPK2 after the yeast ortholog [8] and inositol pentakisphosphate 2-kinases [9], commonly called IPK1, again after the yeast ortholog [8].

**Figure 1.**
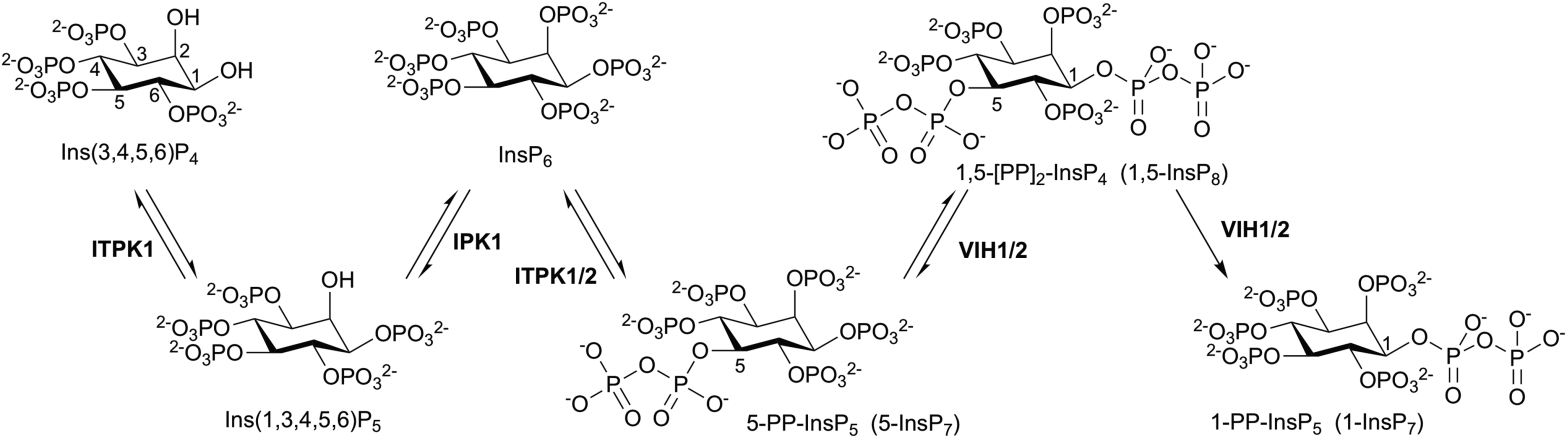
Metabolic relations of inositol phosphates and diphosphoinositol phosphates in plants. For VIH1/2, the reactions shown have been elucidated only on the separated kinase and phosphatase domains of the protein; for ITPK1 and IPK1, the reversible reactions are the property of a single catalytic domain.

ITPK1 controls phosphate homeostasis in Arabidopsis and *Atitpk1* mutants accumulate Ins(3,4,5,6)P_4_ and/or its enantiomer [10]. Ins(3,4,5,6)P_4_ is also the dominant InsP_4_ isomer in *Atipk1* mutants which similarly over-accumulate phosphate [11]. Recently, two groups described phosphoanhydride bond formation on the 5-phosphate of InsP_6_ (synthesis of 5-InsP_7_, 5-PP-InsP_5_) catalyzed by recombinant plant ITPK1 and ITPK2 [12,13]. Others have shown that synthesis of 1,5-InsP_8_ (also known as 1,5-bis-PP-InsP_4_) occurs by 1-phosphorylation of 5-InsP_7_ and is mediated by VIH1 and VIH2 [14] (see Figure 1). Deletion of Vih1 and Vih2 recapitulates constitutive Pi starvation response [15,16]. These works confirm the identity of plant InsP_8_ previously described [14,17]. While it seems likely that the ITPK1 contribution to phosphate homeostasis may lie in the provision of the 5-InsP_7_ precursor of 1,5-InsP_8_, in yeast 5-InsP_7_ is the principal agent of activation of the SPX-dependent polyphosphate polymerase VTC [18].

Nevertheless, the question of which PP-InsPs and InsPs competitively control phosphate homeostasis in plants is compounded for many biological and technical reasons: (1) Plants have multiple SPX-domain proteins [19] with potential discrete and/or overlapping functions in phosphate homeostasis and, generally, poorly defined specificity for binding of inositol and diphosphoinositol phosphates. (2) The relative levels of InsP_7_ and InsP_8_ species revealed by radiolabeling [10,12,17], InsP_7_ greater than InsP_8_, are reversed in recent gel electrophoresis determinations [16]. In the latter study, InsP_8_ levels are substantially greater than InsP_7_ in phosphate replete Arabidopsis. (3) The biochemical activities of enzymes such as ITPK1 and VIH1/2 are not fully defined; for VIH1/2, full-length protein has not been studied, while for both ITPK1 and VIH1/2, the reversibility of the hydroxykinase- and phosphokinase functions of these enzymes is not described.

Here, we focus on ITPK1, disruption of which in Arabidopsis has profound effect on InsP_6_ and InsP_7_ as well as Ins(3,4,5,6)P_4_ [10,20]. To study ITPK1, we employ a suite of accessible methodologies that have not previously been applied to diphosphoinositol phosphates. They afford the opportunity to distinguish known and novel diphosphoinositol phosphates and to study the reversibility of enzymes that catalyze their interconversion. The pathways in plants in which ITPK1 participates and those revealed by this study are shown in Figure 1.

## Materials and methods

### Inositol phosphates

The structures of all inositol phosphates and diphosphoinositol phosphates described in this study are numbered according to the 1d-numbering convention and are shown in Supplementary Figure S1.

InsP_6_ was obtained from Merck Millipore (Product No. 407125). An acid-hydrolysate of phytate (Sigma P-8810) was prepared and used as chromatographic standard according to Madsen et al. [21]. All InsP_5_ isomers used as substrate for ITPK1 were obtained from Sichem as decasodium salts. Ins(1,3,4,5,6)P_5_ obtained from Sichem showed evidence of phosphate migration between *cis*-vicinal hydroxyls. Ins(1,3,4,5,6)P_5_ used as substrate for IPK1 was synthesized according to published procedures [22] and did not show evidence of phosphate migration. InsP_4_s and InsP_3_s were obtained from Cayman Chemical Company or were synthesized according to published procedures [23]. Comparisons made between different enantiomers of enantiomeric pairs were performed with compounds obtained from one source only. Diphosphoinositol phosphates (Supplementary Figure S1): 1-InsP_7_, 3-InsP_7_, 5-InsP_7_ and 5PP-Ins(1,3,4,6)P_4_ were synthesized similarly to published procedures [24]. 4-InsP_7_ and 6-InsP_7_ were synthesized by Dr Henning Jessen, Institute of Organic Chemistry, and the Centre for Integrative Biological Signalling Studies, University of Freiburg, Germany. Acid-catalyzed migration of phosphate on ^32^P-labeled InsPs was performed according to Stephens and Downes [25]. ^32^P-labeled InsPs were used directly for HPLC without processing to remove nucleotides.

### Enzymes: ITPK1

*At*ITPK1 was cloned into the pOPINF plasmid [26], to generate a construct for expression of protein with a 3C cleavable C terminal His tag. Primer sequences used were *5*′*-AAGTTCTGTTTCAGGGCCCG*ATGTCAGATTCAATCCAGGAAAG-3′ and *5*′*-ATGGTCTAGAAAGCTTTA*GACATGATTCTTCTTAGTGAC-3′, where the sequence in italics is specific for recombination with the pOPINF vector. Linearized pOPINF plasmid, digested with HindIII and KpnI, was recombined with the PCR product using the In Fusion HD enzyme kit (Clontech) and subsequently transformed into *Escherichia coli* Stellar cells (Clontech). Colonies were confirmed by PCR amplification and transformed into *E. coli* Rosetta 2(DE3)pLysS (Novagen) for protein expression.

ITPK1:His in pOPINF was expressed at 0.1 mM IPTG in Rosetta2 *E. coli* at 18°C overnight and lysed in 25 mM HEPES pH 7.5, 350 mM NaCl, 1 mM DTT, 20 mM imidazole, 1% triton using a French Pressure Cell. Clarified lysate was loaded onto a 5 ml Ni-NTA column (Qiagen) equilibrated in 25 mM HEPES pH 7.5, 350 mM NaCl, 1 mM DTT, 20 mM imidazole and eluted in the same buffer with 20–250 mM imidazole over a 100 ml gradient. The recombinant protein was subsequently purified using a Superdex 75 10/300 column in 20 mM Tris pH7.5, 200 mM NaCl, 2 mM DTT, 10% glycerol, concentrated and stored at −80°C.

IPK1 was prepared according to Whitfield et al. [27] and assayed under the same conditions as ITPK1.

### Kinase assays

ITPK1 (typically 0.036 µM, with InsP_4_; or 33.6 µM, with InsP_6_) was incubated at 25°C in 10 mM HEPES pH 7.5, 1 mM MgCl_2_ with 1 mM ATP and 0.5 mM or 1 mM inositol phosphate substrate for periods up 2 h. Assays were typically of 50–100 µl volume.

Kinase assays run under regenerating conditions also contained 5 mM phosphocreatine, 3 U creatine kinase and 1 mM ATP under standard conditions. For ATP *K*_m_ calculations ITPK1 was assayed with 0.08–10 mM ATP for 15 min, 40 min or 2 h, depending on the substrate used.

Reactions were terminated by the addition of an equal volume of 60 mM (NH_4_)_2_HPO_4_, pH 3.5, with or without incubation at 95°C for 3 min. Samples were clarified by centrifugation at 14 000×***g*** for 5 min and subsequently diluted with an equal volume of deionized water. The injection volume was typically 50 µl.

For incorporation of ^32^P, assays run in 50 µl volume under ATP-regenerating conditions, 0.5 mM ATP, were supplemented with 10 kBq of [γ-^32^P] ATP 3000 Ci mmol^−1^ (NEG 502A, PerkinElmer). Assays were run for 2 h at 25°C and reactions stopped by freezing at –20°C. Typically, 25 µl aliquots of the reaction products were diluted to 100 µl with water and 50 µl injected.

### HPLC analyses

HPLC was performed according to Whitfield et al. [27] using either 0.6 M methanesulfonic acid or 0.8 M HCl in buffer reservoirs. Inositol phosphates were detected as ferric complexes by UV detection at 290 nm [28]. In some experiments, a second channel of UV data was collected at 254 nm to detect nucleotides. Inositol phosphates are also detected but with reduced sensitivity at this wavelength.

In some experiments, inositol phosphates were analyzed by strong anion exchange high-performance liquid chromatography using a 4.6 × 235 mm Partisphere (Whatman) SAX column according to Kuo et al. [10]. Inositol phosphates were monitored by incorporation of ^32^P, detected by Cerenkov counting in a Radiomatic 515 series Flow Detector (Canberra Packard). The detector was set to an integration interval of 12 s, and a second channel of data was collected from a UV detector placed upstream of the radio-detector and set to 254 nm to monitor nucleotides.

For calculation of kinetic parameters, peak areas of inositol phosphates were integrated with ChromNav v.2 (Jasco). For the reproduction of HPLC profiles, data were exported from ChromNav v.1 or v.2 (Jasco) or Flo-One (Canberra Packard) software as *x*,*y* data and redrawn in GraFit v.7 [29].

## Measurement of absorbance spectra of inositol phosphate ferric complexes

UV spectra were measured for Ins(3,4,5,6)P_4_ and InsP_6_ in 0.4 mM ferric nitrate, 0.4 M methanesulfonic acid, 0.67% (w/v) perchloric acid in quartz glass cuvettes.

### Substrate docking calculations

The ITPK1 from *Entamoeba histolytica* [30] shares 55% sequence identity (60% including conservative substitutions) with the Arabidopsis enzyme when calculated over active site residues alone. For the human enzyme, this rises to 70% identity (80% with conservatively varied substitutions). For this reason, it was decided to build a molecular model for Arabidopsis ITPK1 by reference to the structure of the human enzyme. The amino acid sequence of Arabidopsis ITPK1 (Genbank, At5g16760) was accordingly submitted to Phyre2 [31] and a structural model for the enzyme built by reference to PDB ID: 2QB5 (Crystal Structure of Human Inositol 1,3,4-Trisphosphate 5/6-Kinase (ITPK1) in Complex with ADP and Mn^2+^). Regularization of polypeptide geometry was carried out using Coot [32] where necessary. The final model contained ATP and two magnesium ions. Docking of experimentally verified inositol phosphate substrates (Supplementary Table S1) was carried out using Autodock Vina [33]. A fully flexible model for the ligand and fixed model for the receptor were employed. From the energy-ranked docked conformations (poses) calculated for each substrate, the best ‘productive’ pose was selected on the basis of (i) lowest energy and (ii) positioning of the acceptor site for hydroxykinase (oxygen) or phosphokinase (phosphorus) activity within a maximum of 3.8 Å of the γ-phosphate phosphorus atom of ATP. The cutoff distance of 3.8 Å was chosen to reflect the fact that a rigid model for the protein was employed in the calculation. Productive poses are those that are reasoned to most likely lead to phosphorylation. Poses were rendered in PyMOL (The PyMOL Molecular Graphics System, Schrödinger, LLC.). Docking data and models are provided as Supplemental Information.

Accession Numbers Sequences corresponding to the subjects of this study can be found in the GenBank/EMBL databases under the following accession numbers: ITPK1 (At5g16760), IPK1 (At5g42810).

## Results

We employed anion exchange chromatography on an acid- and hydroxide-stable quaternary amine-functionalized latex (CarboPac PA200, Dionex) as we have done before [27]. This chemistry allows the use of the widest range of eluents. The use of acid allows detection by post-column complexation of inositol phosphate with ferric ion (in acid conditions) and detection of the ferric complex at 290 nm [28]. While the original literature does not explain the physicochemical basis of the absorbance, we may assume from its magnitude (we measured an extinction coefficient of ca. 3200 M^−1 ^cm^−1^ for the complex with InsP_6_) that the absorbance at 290 nm arises from charge transfer transitions, i.e. ligand-field splitting effects of inositol phosphate disturbing symmetry of the octahedral geometry of hexa-co-ordinate hydrated ferric ion [34]. Moreover, while this is a method of choice for analysis of inositol phosphates in animal feed digestive situations where inositol phosphates are abundant [35], we consider that the sensitivity of the method is not widely appreciated for other purposes.

**Figure 2.**
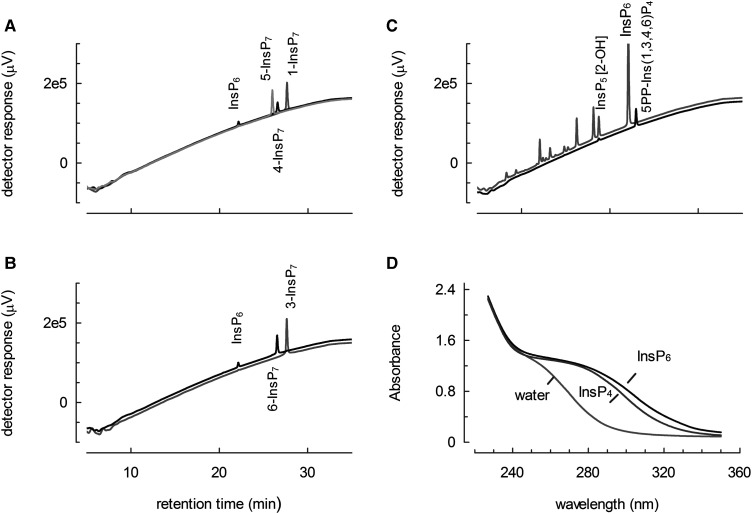
Separations of inositol phosphates and diphosphoinositol phosphates on CarboPac PA200 eluted with HCl. (**A**) InsP_6_, black line; 1-InsP_7_, dark gray line; 5-IP_7_, light gray line and 4-InsP_7_, black line; (**B**) InsP_6_, black line; 3-InsP_7_, dark gray line and 6-InsP_7_, black line; (**C**) an acid-hydrolysate of InsP_6_, with InsP_6_ and Ins(1,3,4,5,6)P_5_ (InsP_5_ [2-OH]) labeled, dark gray line; and 5-PP-Ins(1,3,4,6)P_4_, black line; (**D**) absorbance spectra of ferric HClO_4_ (aq) complexes with InsP_6_, black line; Ins(1,4,5,6)P_4_, dark gray line and water, gray line. These experiments have been repeated more than three times with similar results. The structures of compounds described are shown in Supplementary Figure S1.

We used both methanesulfonic acid and HCl as eluents. Separations with HCl of InsP_6_, 5-InsP_7_, 1-InsP_7_ and 4-InsP_7_ are shown (Figure 2A) and of InsP_6_, 3-InsP_7_ and 6-InsP_7_ (Figure 2B). Enantiomeric pairs, 1-InsP_7_/3-InsP_7_ and 4-InsP_7_/6-InsP_7_ co-eluted (cf. Figure 2A and B), distinct from the *meso*-compound 5-InsP_7_, well resolved from 5-PP-Ins(1,3,4,6)P_4_ which eluted earlier, shortly after InsP_6_ (Figure 2C). The peaks shown represent injections of ca. 1–2 nmol of compound. The 4-InsP_7_ and 6-InsP_7_ samples available to us showed the presence of some InsP_6_ impurities. The absorbance spectra of Ins(1,4,5,6)P_4_ and InsP_6_ complexes of ferric ion, are shown (Figure 2D). A calibration curve of detector response for InsP_6_ is shown for methanesulfonic acid eluent, which gives a flatter baseline, in Supplementary Figure S2. The method is sensitive enough for detection of ca. 50 pmol of InsP_6_ on-column in our hands, but methanesulfonic acid at this concentration did not elute diphosphoinositol phosphates. In measurements of the kinetic parameters of ITPK1 reported later in this manuscript we observed approximately equivalent peak areas for InsP_6_ and 5-InsP_7_ eluted with the stronger acid HCl. The estimations are explained at that juncture.

The opportunity to resolve and measure diphosphoinositol phosphates beside ‘lower’ inositol phosphates without the use of radiolabel is an advance on separations on Partisphere SAX columns, whose silica-based chemistry is not stable under the acid conditions required of post-column complexation with ferric ion. The use of UV detection further allows sampling of data at rates (typically up to 100 Hz) offering resolution beyond fraction collection and scintillation counting, as exemplified [14,36], and beyond on-line radioactivity counting [2]. The method is more convenient and ca. 50–100-fold more sensitive than ^31^P-NMR (as used to verify the InsP_6_ kinase activity of ITPK1, Figure 2, [12]). In our hands, the method is several-fold less sensitive than the metal dye detection-HPLC method of Mayr [37] and is much less compromised by the baseline changes that we have observed of gradients capable of resolving InsP_2_ through to InsP_7_ using that method. Moreover, the method we use requires minimal sample preparation as shown for complex animal digesta matrices [35] and, as we show in the following, can be combined with UV detection at wavelengths allowing simultaneous measurement of nucleotides to follow the reversibility of kinase reactions.

**Figure 3.**
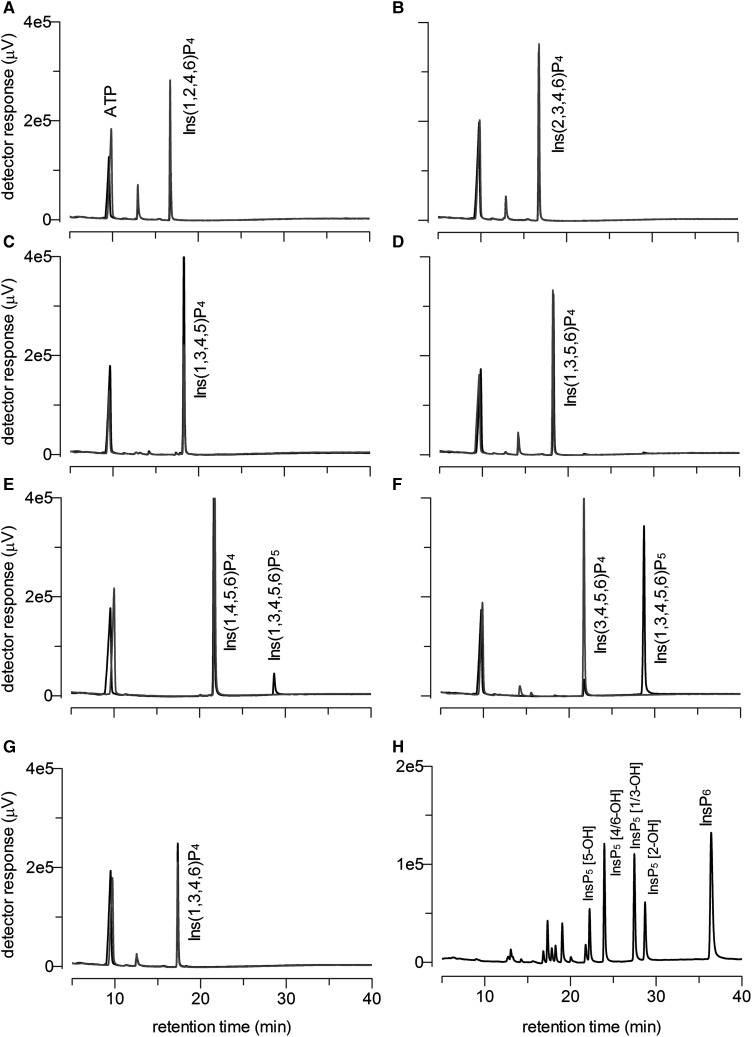
Arabidopsis ITPK1 has robust enantiospecific hydroxykinase activity against Ins(3,4,5,6)P_4_. CarboPac PA200 HPLC analysis of reaction of ITPK1 with (**A**) Ins(1,2,4,6)P_4_; (**B**) Ins(2,3,4,6)P_4_; (**C**) Ins(1,3,4,5)P_4_; (**D**) Ins(1,3,5,6)P_4_; (**E**) Ins(1,4,5,6)P_4_; (**F**) Ins(3,4,5,6)P_4_ and (**G**) Ins(1,3,4,6)P_4_; peaks of substrates (ATP and InsP_4_) and product (Ins(1,3,4,5,6)P_5_) are labeled. Smaller peaks eluting between ATP and InsP_4_ are contaminant InsP_3_s present in the substrate. Chromatograms in panels (A–G) show a no enzyme control, gray trace, and reaction products, black trace. (**H**) shows elution of an hydrolysate of InsP_6_. The HPLC column was eluted with a gradient of methanesulfonic acid. Assays of 50 µl volume were performed in 20 mM HEPES, pH 6.5, 1 mM MgCl_2_, 1 mM ATP, 0.5 mM InsP_5_ in an ATP-regenerating system with 0.036 µM enzyme. These experiments have been repeated more than five times with similar results. The structures of compounds described are shown in Supplementary Figure S1.

We used these methods to examine the substrate specificity of Arabidopsis ITPK1, which belongs to a class of enzyme that has been shown to possess 1-, 5- and 6-hydroxykinase and (InsP_6_) 5-phosphokinase activities. The availability of pairs of enantiomeric substrates, not available in the radiolabeled form, beside *meso*-compounds, allowed analysis of the enantiospecificity of the enzyme. Of particular interest to us were InsP_4_ substrates, since Ins(3,4,5,6)P_4_ is elevated in *itpk1* and *ipk1* mutants that share a common misregulation of phosphate homeostasis [10,11,38] attributed to deregulated diphosphoinositol phosphate synthesis [15,16]. Of the enantiomeric pairs Ins(1,2,4,6)P_4_/Ins(2,3,4,6)P_4_, Ins(1,3,4,5)P_4_/Ins(1,3,5,6)P_4_ and Ins(1,4,5,6)P_4_/Ins(3,4,5,6)P_4_ (all shown in Supplementary Figure S1), only the latter pair were substrates under ATP-regenerating conditions. The enantiomers were phosphorylated on the 3- and 1-positions, respectively (Figure 3A–H). The activity was robust for both enantiomers, but with much greater activity for Ins(3,4,5,6)P_4_ (Figure 3F)_._ The *meso*-isomer Ins(1,3,4,6)P_4_, a canonical substrate of phosphoisomerase activity of *Eh*ITPK1and human ITPK1 [30] was not a substrate for hydroxykinase or phosphoisomerase activity (Figure 3G), consistent also with the lack of Ins(1,3,4,5)P_4_ hydroxykinase or phosphoisomerase activity (Figure 3C).

We also tested many InsP_3_ substrates including the enantiomeric pair Ins(1,4,6)P_3_/Ins(3,4,6)P_3_ (the structures are shown in Supplementary Figure S1 and the HPLC profiles of the reaction products are shown in Supplementary Figure S3A–D). Consistent with the phosphorylation of the enantiomeric pair Ins(1,4,5,6)P_4_/Ins(3,4,5,6)P_4_, ITPK1 showed phosphorylation of Ins(1,4,6)P_3_ at the 3-position and Ins(3,4,6)P_3_ at the 1-position. The presence of an Ins(1,3,4,5)P_4_ peak for all three InsP_3_s tested, possibly indicates phosphoisomerase activity at the level of InsP_4_, under these (non-regenerating for ATP) conditions. The lack of Ins(1,4,5,6)P_4_/Ins(3,4,5,6)P_4_ product for Ins(1,4,6)P_3_ or Ins(3,4,6)P_3_ substrates (Supplementary Figure S3B,C) discounts 5-phosphorylation of these substrates, while the ratio of substrate to product peaks reveals that of the pair, Ins(3,4,6)P_3_ is the preferred substrate.

**Figure 4.**
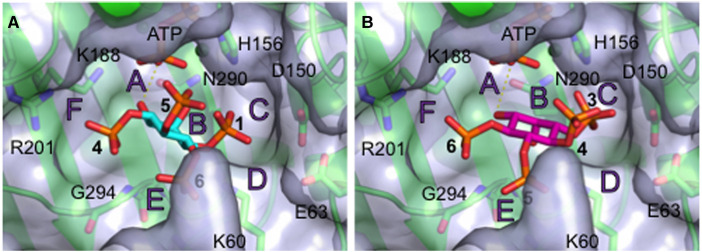
A structural model for enantiospecific kinase activity of *At*ITPK1 towards InsP_4_ substrates. Molecular docking-derived models of the complexes of ITPK1 with ATP/Mg^2+^ and Ins(1,4,5,6)P_4_ (**A**) and Ins(3,4,5,6)P_4_ (**B**). A cartoon representation of ITPK1 is shown in green along with stick representations of ATP and selected active site residues (labeled). The ITPK1 molecular surface is shown in gray. Magnesium ions (partially obscured) are shown as dark green spheres. The distances of closest approach of the γ-phosphate phosphorus of ATP and receiving hydroxyl oxygen (indicated by yellow dashed lines) are less than 3.8 Å in both cases. Specificity subsites as described by Miller et al. [30] are indicated by capital letters (A–F) in each panel. The structures of compounds described are shown in Supplementary Figure S1.

In light of the consistent pattern of phosphorylation of 3- and 1- hydroxyls of Ins(1,4,6)P_3_/Ins(1,4,5,6)P_4_ and Ins(3,4,6)P_3_/Ins(3,4,5,6)P_4_ pairs, respectively, we modeled the binding of the enantiomers Ins(1,4,5,6)P_4_ and Ins(3,4,5,6)P_4_ (to ITPK1 (Figure 4)). We used as reference the specificity subsite nomenclature described for the crystal structure of *E. histolytica* ITPK1 [30]. On this basis our modeling suggests significant interaction of the 4-phosphate of Ins(1,4,5,6)P_4_ and of the 6-phosphate of the Ins(3,4,5,6)P_4_ in site F (Figure 4A,B). Similarly, we posit that the respective 1- and 3- phosphates of Ins(1,4,5,6)P_4_ and Ins(3,4,5,6)P_4_ make contacts in site C. Collectively, residues in these subsites are likely determinants of the reactivity of ITPK1. Indeed, K188A mutation abrogates the activity of ITPK1 against InsP_6_ in *kcs1 vip1 ddp1* yeast, as does D288A mutation [12]. Irrespective of the fine detail, the symmetry-generating capacity of ITPK1's hydroxykinase activity (Supplementary Table S2) suggests fundamental differences in the pose of enantiomeric substrates. We posit that the inositol ring can bind either in ‘obverse’ (axial 2-OH group facing up and pointing out of active site) as presented for the Ins(1,4,5,6)P_4_ (Figure 4A), or ‘reverse’ (2-OH group face down) orientation as presented for Ins(3,4,5,6)P_4_ (Figure 4B).

**Figure 5.**
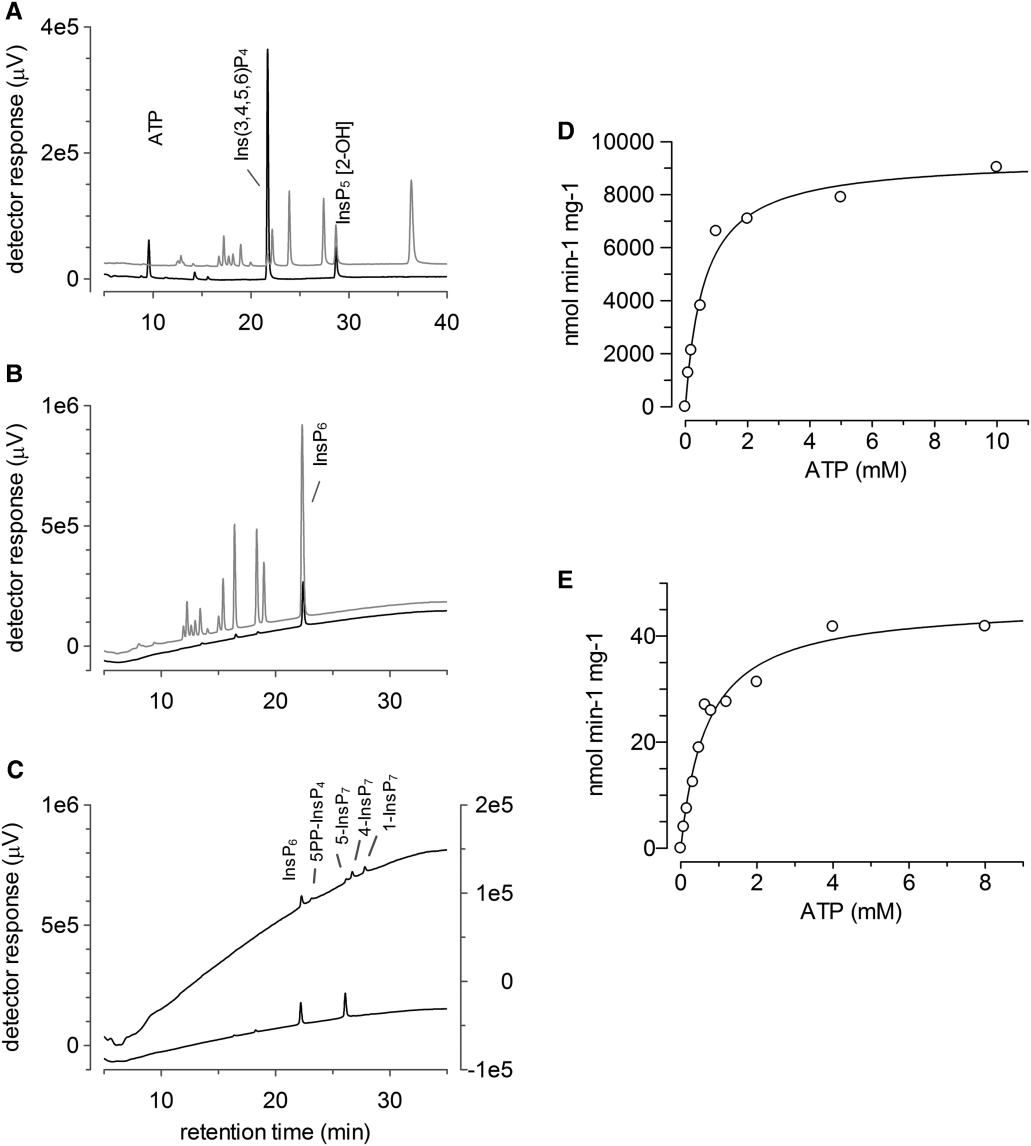
Comparison of Ins(3,4,5,6)P_4_ and InsP_6_ as substrates of Arabidopsis ITPK1. Products of reaction with Ins(3,4,5,6)P_4_ were analyzed with a methanesulfonic acid gradient (**A**), while products of reaction with InsP_6_ under the same conditions were analyzed with an HCl gradient (**B)**; (**C**), as (B) with 100-fold more enzyme. For (A,B), an acid-hydrolysate of InsP_6_ (different amounts) is shown in gray, shifted on the *Y*-axis to aid visualization. For (C), authentic diphosphoinositol phosphates standards are shown in the upper trace (right *Y*-axis). These experiments have been repeated more than five times with similar results. (**D**) Kinetic analysis with Ins(3,4,5,6)P_4_; (**E**) kinetic analysis with InsP_6_; conversion of substrate to product calculated from integrated HPLC peaks. (D,E) data fitted to the Michaelis–Menten equation, each data point is a single measurement from a single experiment. The experiment was repeated three times. *K*_m_ and *V*_max_ (mean and SD) are given in the text. There was a change in eluent batch between runs shown in (A,B) that accounts for the slight shift in retention times. Assays of 50 µl volume were performed in 20 mM HEPES, pH 7.5, 1 mM MgCl_2_, 1 mM ATP, 0.5 mM inositol phosphate in an ATP-regenerating system with 0.036 µM enzyme (A,B,D) or 3.6 µM enzyme and 1 mM substrate. (**E**) Assays with Ins(3,4,5,6)P_4_ were run typically for 20 min, those with InsP_6_ for 40–120 min. The structures of compounds described are shown in Supplementary Figure S1.

Because Ins(3,4,5,6)P_4_ is a known physiological substrate of human ITPK1 [39], we next compared in more detail the relative activity of ITPK1 for Ins(3,4,5,6)P_4_ and the recently identified InsP_6_ substrate. At low enzyme, InsP_6_ was not phosphorylated while Ins(3,4,5,6)P_4_ was (Figure 5A,B). With 100-fold more enzyme, we confirmed the production of 5-InsP_7_ from InsP_6_ (Figure 5C), as reported [12,13]. Because a central tenet of the energy status signaling role of diphosphoinositol phosphates (5-InsP_7_, specifically) is the high (1–1.2 mM) *K*_m_ for ATP of mammalian IP6K [40] and kcs1 [41,42], a more recent estimate for IP6K1 is 0.35 mM [43], we measured kinetic parameters for ATP with these two substrates (Figure 5D,E). Ins(3,4,5,6)P_4_ was, by more than two-orders of magnitude (*V*_max_ 8640 ± 025 nmol min^−1 ^mg^−1^ vs 40 ± 3 nmol min^−1 ^mg^−1^), the stronger substrate. The *K*_m_ ATP values 1.22 ± 0.37 mM and 0.77 ± 0.19 mM for InsP_6_ and Ins(3,4,5,6)P_4_, respectively, identify both InsP_6_ and Ins(3,4,5,6)P_4_ as metabolites that are responsive to energy status, but the much greater activity towards Ins(3,4,5,6)P_4_ focuses attention on this isomer and on historic detailed analyses of inositol phosphate isomerism in plants [2,36,44,45]. Significantly, Ins(3,4,5,6)P_4_ was identified in the duckweed *Spirodela polyrhiza* [36], where it is a substrate for phosphorylation by a cytosolic 1-hydroxykinase [2,46]. The pronounced effect of mutation of ITPK1 on phosphate homeostasis [10] gives physiological context to this activity.

To estimate the sensitivity of the method for detection of diphosphoinositol phosphates, we took advantage of the characterized InsP_6_ kinase activity of ITPK1 to synthesize 5-InsP_7_ from the readily available precursor InsP_6_. We note that the lack of commercial availability of diphosphoinositol phosphates does not allow easy comparison of the chemical stability or purity of different diphosphoinositol phosphates between laboratories, or their batch-to-batch variation, particularly given the difficulty in separation, detection and quantification. Consequently, we set up an assay with sufficient enzyme to effect the substantive conversion of InsP_6_. The data (Supplementary Figure S4A–C) show that for HPLC runs from assays with constant starting InsP_6_, the sum of the integrated peak areas for substrate and product (InsP_6_ + InsP_7_) did not vary substantially despite greater than 50% reduction in InsP_6_ peak area. Thus, we conclude that the sensitivity of the detection of InsP_6_ and 5-InsP_7_ is very similar. Moreover, an example chromatogram (Supplementary Figure S4D) shows that with ease it is possible to detect 5-InsP_7_ at 1% conversion in an assay where approximately one-quarter of the assay products from a 50 µl assay with 1mM InsP_6_ were applied to the column (ca. 125 pmol). Close inspection of the baseline (see inset) makes apparent that the sensitivity of the method is rather better than this, probably approaching 20 pmol. For the purpose of illustrating the utility of our methods for *in vivo* measurements, Supplementary Figure S5 shows example chromatograms from the seeds of Arabidopsis wild type, *ipk1-1* and *mrp5-2* mutants (lines described [10]) and Supplementary Figure S6 shows example chromatograms of InsP_6_ fractions purified from maize and rice bran [21].

**Figure 6.**
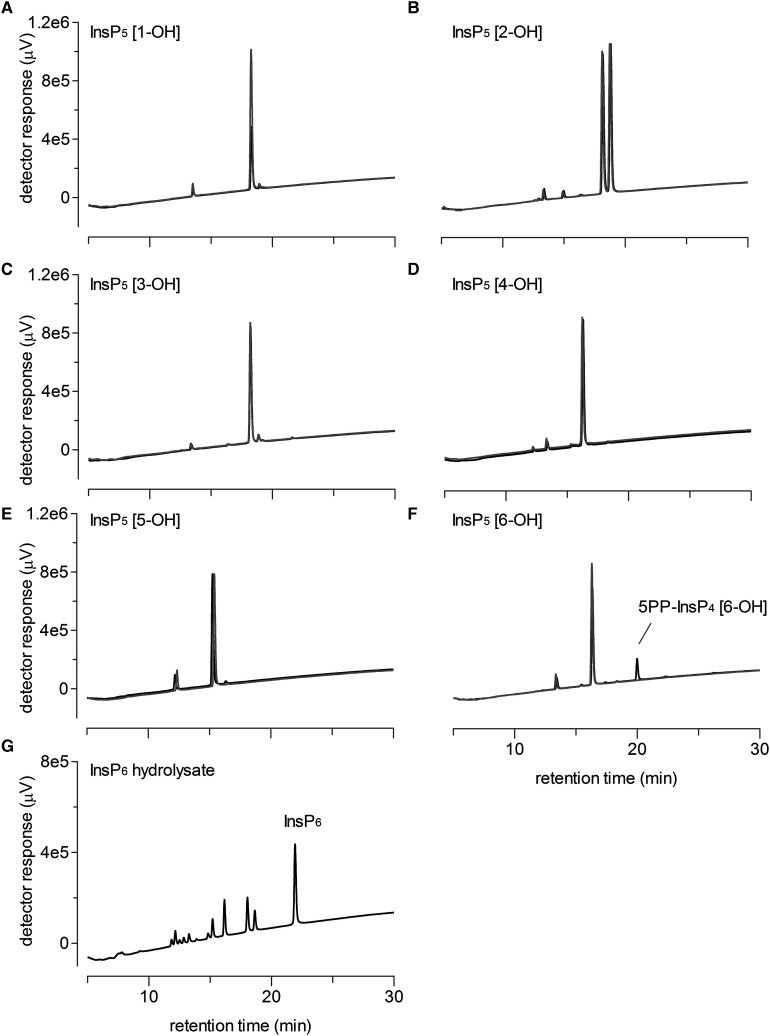
Arabidopsis ITPK1 shows enantiospecific phosphokinase activity on InsP_5_. CarboPac PA200 HPLC analysis of reaction products of ITPK1 with (**A**) Ins(2,3,4,5,6)P_5_, InsP_5_ [1-OH]; (**B**) Ins(1,3,4,5,6)P_5_, InsP_5_ [2-OH]; note the commercial product contains near equimolar InsP_5_ [1/3-OH]; (**C**) Ins(1,2,4,5,6)P_5_; InsP_5_ [3-OH]; (**D**) Ins(1,2,3,5,6)P_5_; InsP_5_ [4-OH]; (**E**) Ins(1,2,3,4,6)P_4_, InsP_5_ [5-OH]; (**F**) Ins(1,2,3,4,5)P_5_, InsP_5_ [6-OH]; (**G**) a set of standards obtained by acid hydrolysis of InsP_6_ is shown. Chromatograms in panels (A–F) show a no enzyme control, gray trace, and reaction products, black trace. Assays of 50 µl volume were performed in 20 mM HEPES, pH 6.5, 1 mM MgCl_2_, 1 mM ATP, 0.5 mM InsP_5_ in an ATP-regenerating system with 0.036 µM enzyme. The HPLC column was eluted with a gradient of HCl. This experiment has been repeated three times with similar results. The structures of compounds described are shown in Supplementary Figure S1.

We also tested whether ITPK1 has activity against Ins(1,3,4,5,6)P_5_ (for structures of InsP_5_s see Supplementary Figure S1). The data of Figure 6 show that Ins(1,3,4,5,6)P_5_ (InsP_5_ [2-OH]), unlike its metabolic neighbors, Ins(3,4,5,6)P_4_ and InsP_6_ [2], is not a substrate. We point out that the particular substrate obtained from Sichem had almost equimolar InsP_5_ [2-OH] and InsP_5_ [1/3-OH] (Figure 6B). The lack of enzymatic product with InsP_5_ [2-OH]/[1/3-OH] is consistent with the lack of secondary products from Ins(1,4,5,6)P_4_ and Ins(3,4,5,6)P_4_] (Figure 3). We suggest that Ins(1,3,4,5,6)P_5_ binds in the same mode as Ins(3,4,5,6)P_4_, but that the axial 2-OH is inappropriately oriented for phosphorylation. Were it to adopt the pose of InsP_6_, we would expect to see a 5-PP-Ins(1,3,4,6)P_4_ product (structure shown in Supplementary Figure S1) which elutes after InsP_6_ (Figure 2). The lack of this activity distinguishes ITPK1 from kcs1 [47] and IP6K, as noted [13].

Ins(1,3,4,5,6)P_5_ is, however, not the only InsP_5_ present in plants, multiple peaks of InsP_5_ have been detected by radiolabeling [2,11,14,36,44,45] and in some cases enantiomers resolved. Because Partisphere SAX columns do not adequately resolve Ins(1,2,3,4,6)P_5_, from Ins(2,3,4,5,6)P_5_ or its enantiomer Ins(1,2,4,5,6)P_5_ [2,36,44,45], we tested the ability of ITPK1 to phosphorylate all isomers of InsP_5_ on the CarboPac PA200 column.

**Figure 7.**
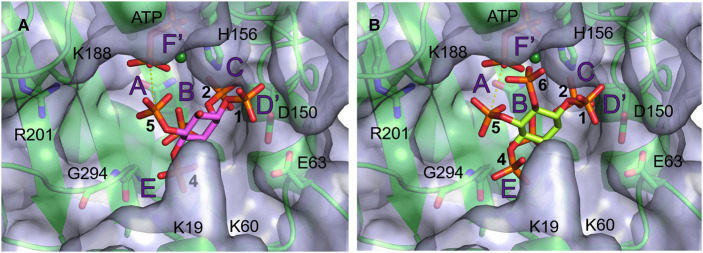
A structural model for phosphokinase activity of ITPK1. A model of the complex of ITPK1 with Ins(1,2,3,4,5)P_5_ and ATP (**A**); a model of the complex of ITPK1 with InsP_6_ and ATP (**B**); speculative contacts between the γ phosphate of ATP and the receiving phosphate of the inositol phosphate substrates (indicated by yellow dashed lines) are less than 3.8 Å in both cases. The model was built on the *human* ITPK1 structure (PDB: 2QB5). The structures of compounds described are shown in Supplementary Figure S1.

InsP_5_ isomers bearing a hydroxyl on the 1-, 2-, 3-, 4- and 5-positions (see Supplementary Figure S1 for structures) were not substrates (Figure 6A–E), but Ins(1,2,3,4,5)P_5_ (InsP_5_ [6-OH]) yielded a novel product that is not InsP_6_ (cf. Figure 6F–G). Given the elution between InsP_5_s and InsP_6_, the most parsimonious explanation is that the product is a novel PP-InsP_4_. We suggest 5-PP-Ins(1,2,3,4)P_4_ (structure shown in Supplementary Figure S1), partly on consideration that neither of Ins(1,2,3,5,6)P_5_ or Ins(1,2,3,4,5)P_5_, nor any of the other InsP_5_ isomers, are phosphorylated on the vacant hydroxyl (Figure 6A–E), but also on account of the discrete retention time, distinct from 5-PP-Ins(1,3,4,6)P_4_ (cf. Figure 2C). We summarize the inositol phosphate hydroxykinase and inositol phosphate phosphokinase reactions catalyzed by ITPK1 in Supplementary Table S2 and present speculative binding modes for Ins(1,2,3,4,5)P_5_ and InsP_6_ to ITPK1 in Figure 7A,B.

The two ligands adopt similar poses in their predicted low energy complexes: binding modes quite dissimilar to those seen for InsP_3_ or InsP_4_ ligands. Specifically, for InsP_6_, the axial 2-phosphate occupies site C and equatorial 3-phosphate site B of the canonical specificity subsite set described by Miller et al. [30]. However, for both Ins(1,2,3,4,5)P_5_ and InsP_6_ ligands the inositol ring rotates to lie roughly perpendicular to that seen for docked InsP_3_ and InsP_4_ ligands and the 4-phosphate consequently occupies site E. This forces the 5-phosphate to occupy site A, the site of phosphotransfer. The 1- and 6-phosphates (where they exist) occupy new sites which we name D′ and F′, respectively. Subsite D′ involves residues Asp63, Asp150 and His156, whilst F′ involves His156 and Ser225. These predicted subsites presumably play a role in helping to ameliorate the problems arising from the need to accommodate the steric bulk and phosphate crowding of these substrates.

The commercial unavailability of PP-InsPs, especially PP-InsP_4_s for which there are 30 theoretical possibilities, five for each InsP_5_ ‘parent’, does not allow facile identification of the novel ITPK1 product on chromatographic grounds. The near micromole quantities required for NMR analysis preclude easy confirmation of identity.

Nevertheless, the identification of predominant Ins(3,4,5,6)P_4_ 1-hydroxykinase activity for ITPK1 affords explanation of *itpk1* phenotype (disruption of InsP_6_ synthesis and accumulation of Ins(3,4,5,6)P_4_ [10,20]). Because ITPK1 and IPK1 individually control phosphate homeostasis, individually accumulate Ins(3,4,5,6)P_4_ [10,11,38] and share substrates and products, we next tested whether ITPK1 and IPK1 constitute an ATP-responsive metabolic cassette linking Ins(3,4,5,6)P_4_ to 5-InsP_7_. We combined the two enzymes with nucleotide and Ins(3,4,5,6)P_4_. We added equimolar ITPK1 and IPK1 and either buffered ATP with an ATP-regenerating system (this maintains high ATP: ADP ratio) or excluded the ATP-regenerating system, varying ATP: ADP ratio.

**Figure 8.**
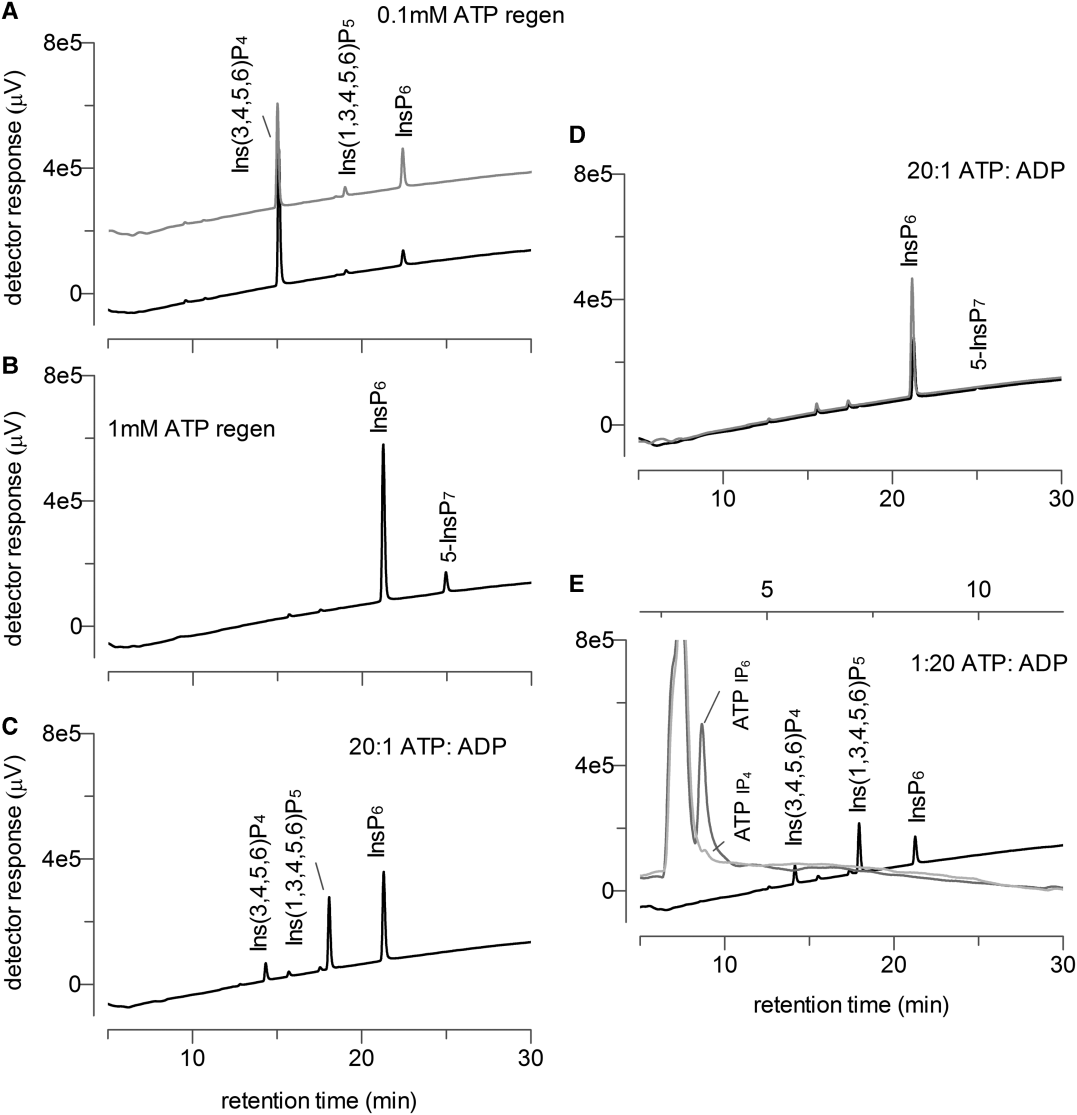
ITPK1 and IPK1 comprise an energy-responsive metabolic cassette. Products of reaction coupled with the two enzymes were analyzed by CarboPac PA200 HPLC. In an ATP-regenerating system at low ATP, (**A**) Ins(3,4,5,6)P_4_ is converted via Ins(1,3,4,5,6)P_5_ to InsP_6_, 20 min reaction, black line; 40 min reaction, gray line; (**B**) with higher ATP, Ins(3,4,5,6)P_4_ is converted via InsP_6_ to 5-InsP_7_; (**C**) in the absence of the regenerating system and at a 20 : 1 ATP:ADP ratio, Ins(3,4,5,6)P_4_ is converted via Ins(1,3,4,5,6)P_5_ to InsP_6_. For (A–C), the purity of the Ins(3,4,5,6)P_4_ substrate used is evidenced in Figure 3F; (**D**) under the same conditions, InsP_6_ is not substantially converted to 5-InsP_7_, no enzyme control, gray trace; reaction with enzyme, black trace; (**E**) at a 1 : 20 ATP:ADP ratio, InsP_6_ is converted via Ins(1,3,4,5,6)P_5_ to Ins(3,4,5,6)P_4_ with concomitant generation of ATP, the additional traces (time, upper *X*-scale; UV_254_, *Y*-scale) show generation of an ATP peak when assays are conducted with InsP_6_ (dark gray) and the absence of this peak when the same experiment is conducted with Ins(3,4,5,6)P_4_ (light gray). Assays of 50 µl or 100 µl volume were performed in 20 mM HEPES, pH 7.5, 1 mM MgCl_2_, with 0.5 mM Ins(3,4,5,6)P_4_ or InsP_6_ and an ATP-regenerating system with 0.1 mM ATP (A); 1 mM ATP (B); or without the regenerating system and 5 mM ATP, 0.25 mM ADP (C,D); or 0.25 mM ATP, 5 mM ADP (E). The enzyme concentration was 0.030 µM ITPK1, 0.014 µM IPK1 for (A,B) and 3.6 µM ITPK1, 3.6 µM IPK1 for (C–E). This experiment has been repeated more than three times with similar results; and for (D), the purity of the InsP_6_ substrate is evidenced in Figure 5B. The structures of compounds described are shown in Supplementary Figure S1. There was a change in eluent buffer between the experiments shown in (A,B), accounting for the change in the retention time of the InsP_6_ peak.

In the ATP-regenerating system with 0.1 mM ATP, Ins(3,4,5,6)P_4_ was converted via Ins(1,3,4,5,6)P_5_ to InsP_6_ (Figure 8A) and at 1 mM ATP in the regenerating system to 5-InsP_7_ (Figure 8B). Without the regenerating system, but with 20 : 1 ATP: ADP ratio, Ins(3,4,5,6)P_4_ was robustly converted via Ins(1,3,4,5,6)P_5_ to InsP_6_ (Figure 8C), while InsP_6_ was not significantly phosphorylated (Figure 8D). These experiments show that combination of ITPK1 and IPK1 drives 5-InsP_7_ synthesis from Ins(3,4,5,6)P_4_ in an ATP-dependent manner.

The reversibility of IPK1 [9,48], kcs1 [41] and IP6K [40,49], led us to test the reversibility of ITPK1. With ITPK1 and IPK1 combined at a 1 : 20 ATP: ADP ratio, InsP_6_ was converted via Ins(1,3,4,5,6)P_5_ to Ins(3,4,5,6)P_4_ and/or Ins(1,4,5,6)P_4_ with concomitant generation of ATP (Figure 8E).

**Figure 9.**
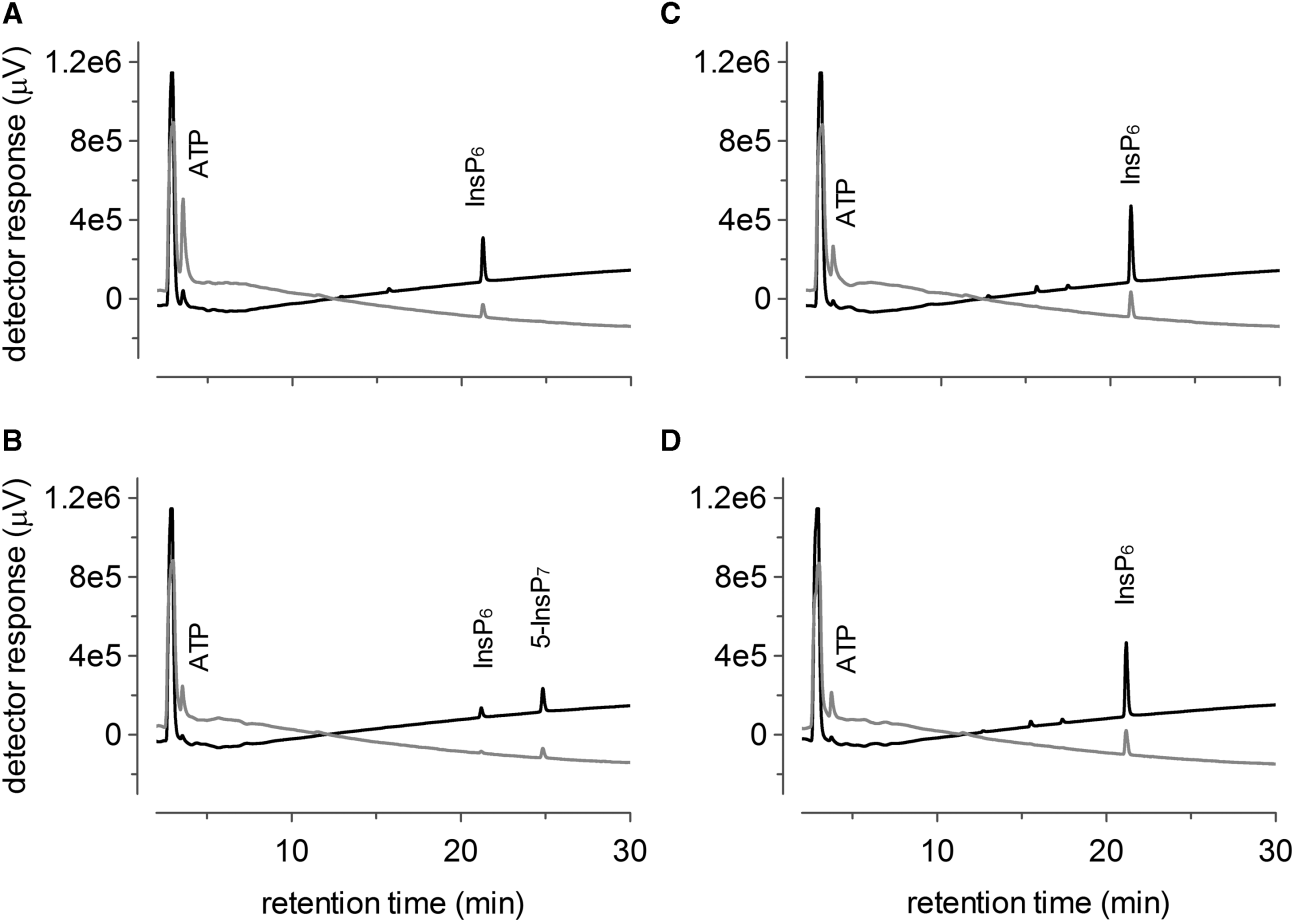
Arabidopsis ITPK1 shows symmetry conserving 5-InsP_7_-ADP phosphotransferase activity. CarboPac PA200 HPLC analysis (HCl gradient) of reaction products of ITPK1 with (**A**), 5-InsP_7_; (**B**) no enzyme control for (A); note conversion of 5-InsP_7_ to InsP_6_ (black trace) with concomitant generation of ATP (gray trace, UV_254_); (**C**) InsP_6_; (**D**) no enzyme control for (C). Assays of 100 µl volume were performed in 20 mM HEPES, pH 7.5, 1 mM MgCl_2_, 0.25 mM ATP, 5 mM ADP, 0.25 mM 5-InsP_7_ or 0.5 mM InsP_6_ with 3.6 µM enzyme for 1 h (A,B) or 3 h (C,D). This experiment has been repeated more than three times with similar results. The structures of compounds described are shown in Supplementary Figure S1.

At the same nucleotide ratio, ITPK1 generated InsP_6_ alone from 5-InsP_7_ with the concomitant production of ATP (Figure 9A,B), but was without effect on InsP_6_ under the same conditions (Figure 9C,D). The sensitivity of the direction of the reaction catalyzed by ITPK1 to nucleotide ratio is in part similar to IP6K, but unlike for IP6K [49], we did not observe phosphotransfer from IP_6_ to ADP, that function is efficiently performed by IPK1 [9,48,50].

**Figure 10.**
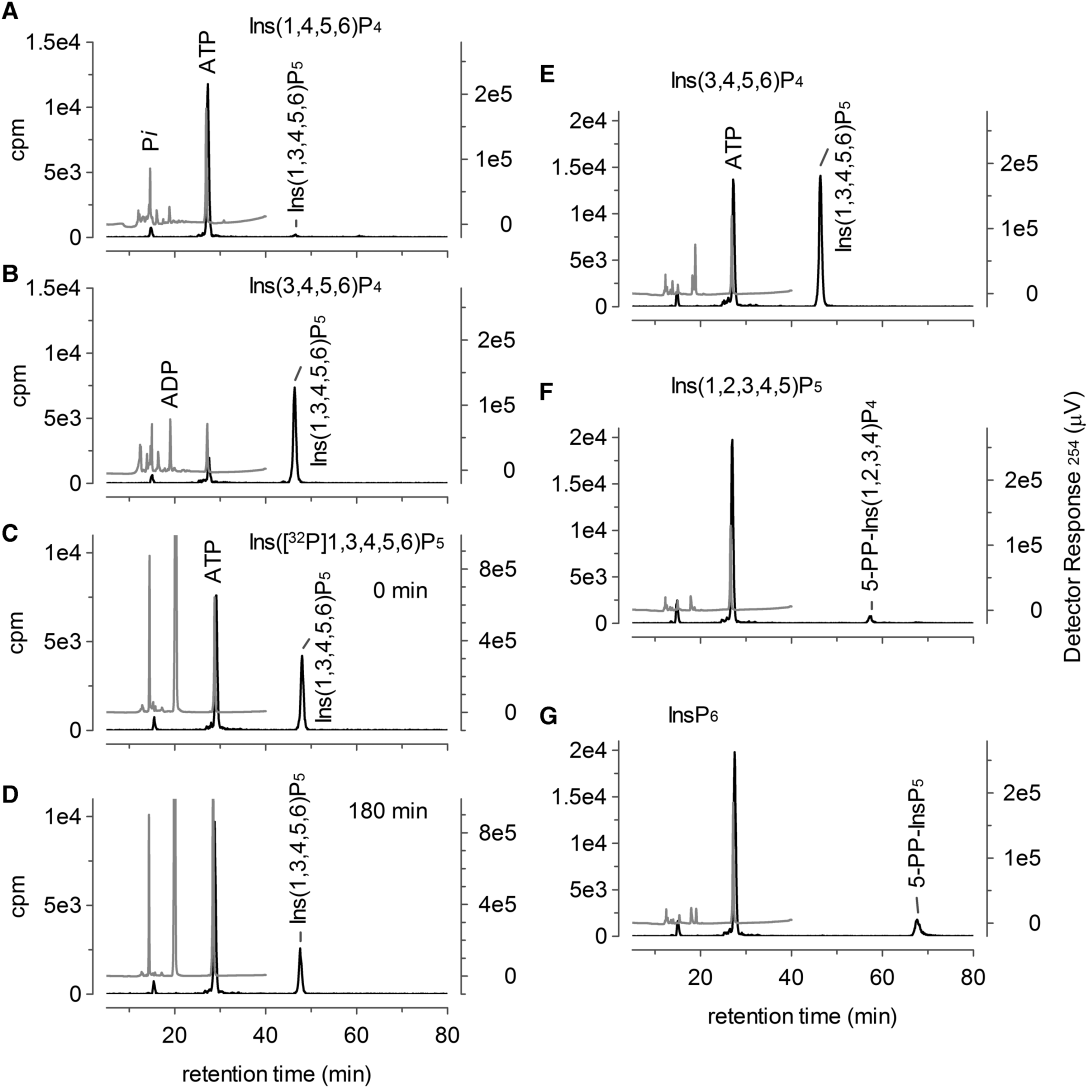
Partisphere SAX HPLC analysis of phosphotransfer reactions catalyzed by ITPK1. Kinase reaction of ITPK1 with [γ-^32^P]ATP and (**A**) Ins(1,4,5,6)P_4_; (**B**) Ins(3,4,5,6)P_4_; ATP synthase reaction of ITPK1 with Ins([^32^P]1,3,4,5,6)P_5_ and ADP (**C**), 0 min; (**D**) 180 min. Comparison of kinase reaction of ITPK1 with Ins(3,4,5,6)P_4_ (**E**); Ins(1,2,3,4,5)P_5_ (**F**) and InsP_6_ (**G**). (A–G) Left *Y*-axis, cpm, black lines; right *Y*-axis, gray trace, UV_254_. All panels show the presence of unconsumed ATP in the assay products. For (D), there is an enzyme-dependent increase in ATP at the expense of Ins([^32^P]1,3,4,5,6)P_5_. Assays in the kinase direction were performed under ATP-regenerating conditions with 1 mM ATP, 0.25 mM inositol phosphate and 0.036 µM enzyme for 1 h (A,B). A separate assay run for 3 h is shown (E–G). Assays in (C,D) were performed with 0.25 mM ATP, 5 mM ADP for 2 h. The structures of compounds described are shown in Supplementary Figure S1.

By labeling of kinase reaction products with [γ-^32^P] (see Supplementary Figure S1) and use of more common Partisphere SAX HPLC, we determined the enantiospecificity of ITPK-mediated dephosphorylation of Ins(1,3,4,5,6)P_5_. First, we synthesized Ins([^32^P]1,3,4,5,6)P_5_, and Ins(1,[^32^P]3,4,5,6)P_5_, from Ins(3,4,5,6)P_4_ and Ins(1,4,5,6)P_4_, respectively (Figure 10A,B). Again, we confirmed that Ins(3,4,5,6)P_4_ is the much stronger substrate. Next we incubated Ins([^32^P]1,3,4,5,6)P_5_ with ITPK1 at unlabeled nucleotide ratio (ATP:ADP, 1 : 20) favoring phosphotransfer to ADP. We did not observe the production of [^32^P]InsP_4_, despite synthesis of [^32^P]ATP and an increase in ATP peak area (in the UV_254_ channel). Significantly, the reaction did not generate orthophosphate product (Figure 10C,D). These data show that ITPK1 has a preferential reversible Ins(3,4,5,6)P_4_ 1-kinase /Ins(1,3,4,5,6)P_5_ 1-phosphotranferase to ADP (ATP synthase) activity, i.e. the phosphorylation of the preferred enantiomeric (asymmetric) substrate to *meso*- (symmetric) product is reversible in generating the same enantiomer of InsP_4_. Using this more common HPLC method we were also able to compare the efficiency of phosphorylation of Ins(3,4,5,6)P_4_, Ins(1,2,3,4,5)P_5_ and InsP_6_ substrates (Figure 10E–G). Ins(3,4,5,6)P_4_ was, again, much the preferred substrate, with Ins(1,2,3,4,5)P_5_ a slightly weaker substrate than InsP_6_. In Supplementary Figure S7, we show that the presumed 5-PP-Ins(1,2,3,4)P_4_ product of phosphorylation of Ins(1,2,3,4,5)P_5_ elutes between Ins(2,3,4,5,6)P_5_ (InsP_5_ [1-OH]) and InsP_6_ on Partisphere SAX HPLC.

## Discussion

Despite historic description of inositol phosphates more highly charged than InsP_6_ in amoeboid organisms [51,52], animals [53,54] and plants [44,55,56], the identity of inositol phosphate kinases in plants capable of forming phosphoanhydride bonds has until recently proved enigmatic [12,13]. Because diphosphoinositol phosphates have garnered attention as a cellular signal of eukaryotic energy status, reviewed [42,57,58], the recent works [12,13] focus attention on 5-InsP_7_ as an agent of energy signaling in plants.

By measuring *K*_m_ ATP for Arabidopsis ITPK1 we show that diphosphoinositol phosphate synthesis from Ins(3,4,5,6)P_4_ is responsive to nucleotide ratio, which taking account of AMP is encapsulated as a metabolic concept in the term energy charge [59]. The low affinity of InsP_6_ kinase for ATP [40] is a central tenet of the signaling role of 5-InsP_7_. Similarly, the *K*_m_ ATP of Arabidopsis ITPK1 (1.2 mM) poises the activity of this enzyme within the estimated physiological range of this nucleotide in the cytosol of plants [60–62]. A logical extension is that the physiological levels of all ITPK1 substrates and products, including Ins(3,4,5,6)P_4_ and Ins(1,3,4,5,6)P_5_, InsP_6_ and 5-InsP_7_, and for that matter Ins(1,2,3,4,5)P_5_ and the presumed 5PP-Ins(1,2,3,4)P_4_, are responsive to energy status. Here, by defining the reversibility of ITPK1 we show that the extent and direction of flux between Ins(3,4,5,6)P_4_ and 5-InsP_7_ is responsive to nucleotide ratio (Figure 1).

While 5-InsP_7_ is a reported regulator of phosphate homeostasis in yeast [63], it has been shown in metazoans, the HCT116 cell line, that both 5-InsP_7_ and 1,5-InsP_8_ are responsive to extracellular Pi, but 1,5-InsP_8_ more so [64]. This is explained in part by inhibition of phosphatase activities of PPIP5K1 and PPIP5K2 by Pi, and activation, for PPIP5K2 of kinase activity.

The plant orthologs of PPIP5K have proved recalcitrant to study as full-length enzymes, but plants bearing deletion of Vih1 and Vih2 show constitutive Pi starvation responses recapitulating itpk1 phenotype [10,15]. The activities of the separated recombinant kinase and phosphatase domains of VIH1 and VIH2 [13–16] are shown (Figure 1). What is not clear is how full-length VIH is influenced by different nucleotides or nucleotide ratio *in vitro* or *in vivo*. Since recombinant full-length ScVip1 converts 5-InsP_7_ to InsP_8_ at supra-mM Mg^2+^-ATP and produces InsP_6_ at sub-mM Mg^2+^-ATP [15] we may expect VIH1/2 to do the same, but whether VIH1/2 (or ScVip) show diphosphoinositol phosphate-‘driven’ ATP synthase activity (from InsP_7_ or InsP_8_) in the manner of IP6K and Kcs1 [40,41,49] and *At*ITPK1 (this study) is not resolved. Clearly, the example of *At*ITPK1 illustrates how reversibility of the activity(s) of full-length VIH1/2 is critical to our understanding of phosphate homeostasis, since between them ITPK1 and VIH1/2 set the balance between 5-InsP_7_ and 1,5-InsP_8_ as ligands of SPX-domain-containing proteins.

Reversibility of plant inositol phosphate (hydroxyl) kinases is, however, well documented for ITPKs [6,65] and IPK1 [9,48], the latter echoing earlier work on a mung bean activity [50] for which physiological context in germinative ATP synthesis was predicted as early as 1963 [66]. Consideration of these works shows how enzymes such as IPK1 with large equilibrium constants [9] can drive phosphoanhydride formation. Moreover, they show that phosphoanhydride formation is not a priori a facet of ‘high-energy’ status of either substrate, it can for reversible enzymes merely reflect prevailing substrate concentration.

Considering *At*ITPK1's contribution to InsP and PP-InsP metabolism, Saiardi and co-workers [67] have shown by heterologous expression of ITPK1 orthologs in yeast how inositol phosphate metabolism may be cryptic, or at least hidden to conventional radiolabeling. Without measurement of specific radioactivity of metabolite pools, labeling studies run the risk of missing these ‘cryptic’ pathways. The work of Stephens and Downes [25] is a notable exception that remarkably defined a pathway for phosphorylation of Ins(3,4,6)P_3_ via Ins(3,4,5,6)P_5_ to Ins(1,3,4,5,6)P_5_ that is paralleled in duckweed [2,36,46]. Given the identification of InsP_7_ and InsP_8_ in duckweed [55], it is likely that InsP_8_ synthesis therein is catalyzed through the contribution of orthologs of *At*ITPK1 and *At*IPK1 whose reversible nature in 5-InsP_7_ synthesis/turnover is revealed here (Figure 1).

Moreover, our analysis suggests that ITPK1's substantially greater activity for Ins(3,4,5,6)P_4_ over InsP_6_, coupled through IPK1, is ‘matched’ to the different pool sizes of Ins(3,4,5,6)P_4_ and InsP_6_ [10]. Indeed, InsP_6_ is typically two orders of magnitude more strongly labeled, but not in vegetative duckweed [2,36]. While we caution again of the limitations of radiolabeling without independent measurement of pool size, such a proposition explains well the phenotype of *itpk1* mutants, viz. increase in Ins(3,4,5,6)P_4_ and decrease in 5-InsP_7_.

Most interestingly, perhaps, our work reveals how evolution has assigned the same function, an eminently reversible and nucleotide-sensitive InsP_6_/5-InsP_7_ phosphotransferase activity, to wholly different enzymes in plants and mammals. ITPK1, and perhaps ITPK2, assume the role of IP6K, or vice-versa, despite the presence of ITPK1 homolog in mammals. This places special emphasis on ITPK1. As we have shown, *itpk1* mutants make less InsP_6_, InsP_7_ and, most likely, InsP_8_, and hyperaccumulate phosphate [10]. Others [15,16] have collectively assigned special function to InsP_8_ as the cognate binding partner for SPX1 which interacts with the master transcriptional regulator, PHR1, of phosphate starvation responses. Consistent with studies in barley leaves [68], we have previously shown a shoot-specific near doubling in ATP levels on phosphate starvation. This increase, also in ATP/AMP ratio, was associated with a shoot-specific increase in InsP_7_. This is consistent with the nucleotide ratio-dependent control of ITPK1 that we demonstrate here. Others, however, have reported that whole seedlings starved of phosphate, and hence showing full phosphate starvation responses, increase nucleotides and ATP/ADP ratio approximately 2-fold on re-supply of phosphate [15].

Of course, plants are photosynthetic autotrophs, which yeast and mammals are not. Plants couple phosphate import across the chloroplast membrane to export of triose phosphate for growth, in an obligatory manner. These points caution that there are substantive differences in phosphate homeostasis between eukaryotes, extending to the engagement of multiple SPX-domain proteins in plants [19]. Just as there are substantive differences in the fundaments of phosphoinositide signaling between plants, animals and yeast we may anticipate differences in control of phosphate homeostasis at the level of diphosphoinositol phosphate interaction with SPX proteins. The evolutionary diversification of ITPK [6,10,12,13,65] and SPX [19] families in plants say as much. Significantly, we have shown that ITPK2, despite its InsP_6_ kinase activity [12,13] does not regulate phosphate starvation responses, nor do ITPK3 and ITPK4 [10]. If plants engage diphosphoinositol phosphates in as wide a range of physiological functions as animal cells, we can expect ITPK1 and ITPK2 to be engaged in other aspects of plant physiology, but for plants — integration with photosynthesis is likely to be critical. Here, tools that allow simultaneous measurement of subcellular nucleotide pools [62] could be brought to bear to elucidate the complex relationships between intracellular phosphate, nucleotides and diphosphoinositol phosphates. Nevertheless, the responsiveness of the direction of ITPK1 activity to the nucleotide ratio is likely a critical control point.

## Open access

Open access for this article was enabled by the participation of University of East Anglia in an all-inclusive *Read & Publish* pilot with Portland Press and the Biochemical Society under a transformative agreement with JISC.

## Abbreviations

*At*IPK1: *Arabidopsis thaliana* inositol pentakisphosphate 2-kinase *At*ITPK1, *Arabidopsis thaliana* inositol tris/tetrakisphosphate kinase 1
*Dd*ITPK1: *Dictyostelium discoideum* inositol tris/tetrakisphosphate kinase 1
DTT: reduced dithiothreitol
EDTA: ethylenediamine tetra-acetic acid
HEPES: 4-(2-hydroxyethyl)-1-piperazineethane sulfonic acid
His: histidine
HPLC: high-pressure liquid chromatography
*Hs*ITPK1: *Homo sapiens* inositol tris/tetrakisphosphate kinase 1
IP6K: inositol hexakisphosphate kinase
IPK2: inositol polyphosphate multikinase
Ins(1,3,4)P_3_: 1d-*myo*-inositol 1,3,4-trisphosphate
Ins(1,4,6)P_3_: 1d-*myo*-inositol 1,4,6-trisphosphate
Ins(3,4,6)P_3_: 1d-*myo*-inositol 3,4,6-trisphosphate
Ins(1,2,4,6)P_3_: 1d-*myo*-inositol 1,2,4,6-tetrakisphosphate
Ins(1,3,4,6)P_4_: *myo*-inositol 1,3,4,6-tetrakisphosphate
Ins(1,4,5,6)P_4_: 1d-*myo*-inositol 1,4,5,6-tetrakisphosphate
Ins(1,3,5,6)P_4_: 1d-*myo*-inositol 1,3,5,6-tetrakisphosphate
Ins(2,3,4,6)P_4_: 1d-*myo*-inositol 2,3,4,6-tetrakisphosphate
Ins(3,4,5,6)P_4_: 1d-*myo*-inositol 3,4,5,6-tetrakisphosphate
Ins(1,2,3,4,5)P_5_: 1d-*myo*-inositol 1,2,3,4,5-pentakisphosphate
Ins(1,2,3,4,6)P_5_: *myo*-inositol 1,2,3,4,6-pentakisphosphate
Ins(1,2,3,5,6)P_5_: 1d-*myo*-inositol 1,2,3,5,6-pentakisphosphate
Ins(1,2,4,5,6)P_5_: 1d-*myo*-inositol 1,2,4,5,6-pentakisphosphate
Ins(1,3,4,5,6)P_5_: *myo*-inositol 1,3,4,5,6-pentakisphosphate
Ins(2,3,5,4,6)P_5_: 1d-*myo*-inositol 2,3,4,5,6-pentakisphosphate
InsP_6_, Ins(1,2,3,4,5,6)P_6_: *myo*-inositol 1,2,3,4,5,6-hexakisphosphate
5-PP-Ins(1,2,3,4)P_4_: 1d-5-diphospho-*myo*-inositol 1,2,3,4-tetrakisphosphate
5-PP-Ins(1,3,4,6)P_4_ 5-diphospho-*myo*-inositol 1,3,4,6-tetrakisphosphate;1-InsP_7_, 1-PP-InsP_5_: 1d-1-diphospho-*myo*-inositol 2,3,4,5,6-pentakisphosphate
3-InsP_7_, 3-PP-InsP_5_: 1d-3-diphospho-*myo*-inositol 1,2,4,5,6-pentakisphosphate
4-InsP_7_, 4-PP-InsP_5_: 1d-4-diphospho-*myo*-inositol 1,3,5,6-pentakisphosphate
5-InsP_7_, 5-PP-InsP_5_: 5-diphospho-*myo*-inositol 1,2,3,4,6-pentakisphosphate
6-InsP_7_, 6-PP-InsP_5_: 1d-6-diphospho-*myo*-inositol 1,2,3,4,5-pentakisphosphate
1,5-InsP_8_: 1d-1,5-bis-diphospho-*myo*-inositol 2,3,4,6-tetrakisphosphate
Ni-NTA: nickel-nitriloacetic acid
*Os*ITPK1: *Oryza sativa* inositol tris/tetrakisphosphate kinase 1
PCR: polymerase chain reaction
PHR1: *Arabidopsis* Phosphate Starvation Response 1
PPIP5K: diphosphoinositol pentakisphosphate kinase
SPX1: SYG1/Pho81/XPR1 domain-containing protein 1
Tris: tris(hydroxymethyl)aminomethane
VIH1: *Arabidopsis thaliana* diphosphoinositol pentakisphosphate kinase 1
VIH2: *Arabidopsis thaliana* diphosphoinositol pentakisphosphate kinase 2

## Supplementary Material

Supplementary Figures S1-S7 and Tables S1-S2

Supplementary Docking files
