# Supplementary material for: An ATP-responsive metabolic cassette comprised of inositol tris/tetrakisphosphate kinase 1 (ITPK1) and inositol pentakisphosphate 2-kinase (IPK1) buffers diphosphosphoinositol phosphate levels
Source: Biochem J. 2020 Jul 24;477(14):2621–38. doi: 10.1042/BCJ20200423 (PMC7115839; doi:10.1042/BCJ20200423)
Supplement: Supplementary Figures S1-S7 and Tables S1-S2 [file BCJ-477-2621-s1.pdf]

Supplementary Information

Figure S1. Structures of compounds described

Figure S2. Sensitivity of detection of $InsP_6$ on MeSA gradients

Figure S3. *At*ITPK1 hydroxykinase activity against $InsP_3$s

Figure S4. Sensitivity of detection of $InsP_6$ and 5-$InsP_7$ on HCl gradients

Figure S5. Inositol phosphate profiles of Arabdidopsis seeds

Figure S6. Inositol phosphates in maize and rice bran $InsP_6$ preparations

Figure S7. Partisphere SAX HPLC profiles of *At*ITPK1 products including PP-InsPs

Table S1. Summary of docking results

Table S2. Reactions catalyzed by ITPK1 tested in this study

Docking files:

Human ITPK1 template	c2qb5BATPMg.pdb
D-Ins(1,4,6)P_3	146IP3.pdb
D-Ins(3,4,6)P_3	346IP3.pdb
D-Ins(1,4,5,6)P_4	1456IP4.pdb
D-Ins(3,4,5,6)P_4	3456IP4.pdb
D-Ins(1,2,3,4,5)P_5	12345IP5.pdb
$InsP_6$	IP6.pdb

Figure S1. Structures of inositol phosphates and diphosphoinositol phosphates described in this study. Compounds marked with an asterisk are novel substrates/products of ITPK1. 5-PP-Ins(1,2,3,4)P_4 is the presumed product of pyrophosphorylation of Ins(1,2,3,4,5)P_5. Compounds are shown with 1D-numbering.

Fig. S1

Inositol trisphosphates

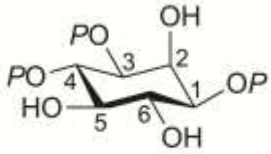

$Ins(1,3,4)P_3$

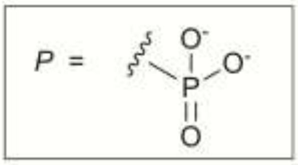

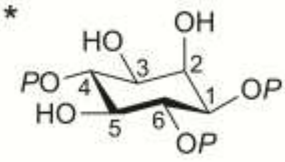

$Ins(1,4,6)P_3$

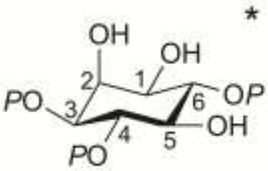

$Ins(3,4,6)P_3$

Inositol tetrakisphosphates

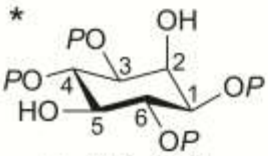

$Ins(1,3,4,6)P_4$

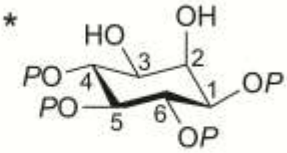

$Ins(1,4,5,6)P_4$

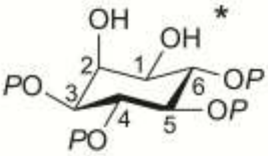

$Ins(3,4,5,6)P_4$

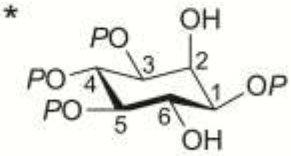

$Ins(1,3,4,5)P_4$

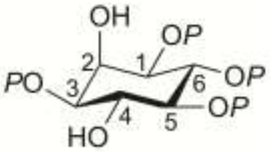

$Ins(1,3,5,6)P_4$

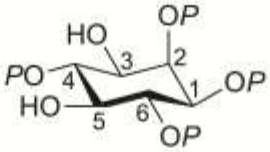

$Ins(1,2,4,6)P_4$

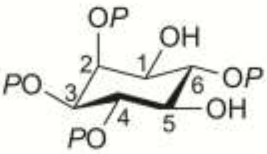

$Ins(2,3,4,6)P_4$

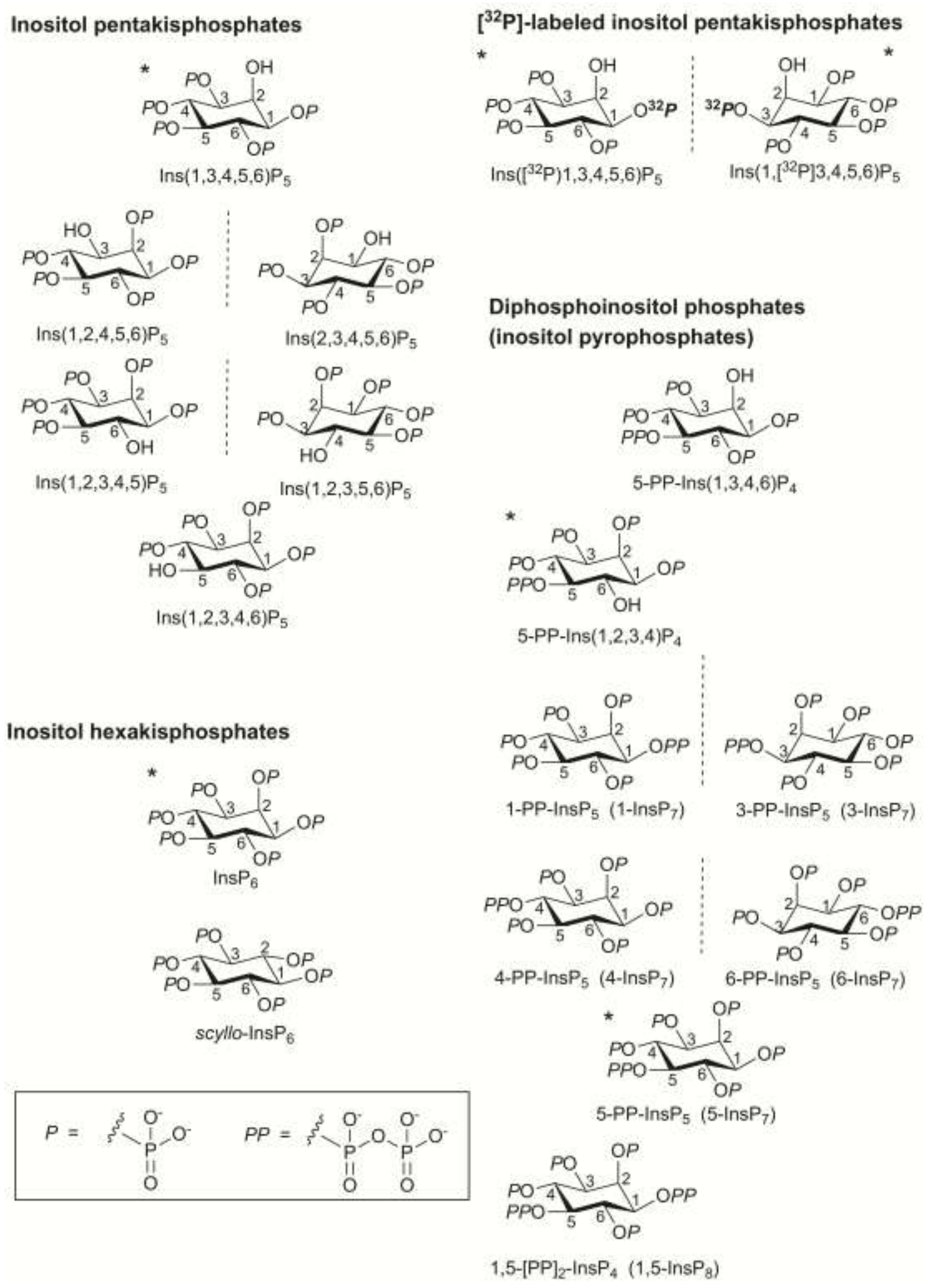
Inositol pentakisphosphates
Ins(1,3,4,5,6)P5
Ins(1,2,4,5,6)P5
Ins(2,3,4,5,6)P5
Ins(1,2,3,4,5)P5
Ins(1,2,3,5,6)P5
Ins(1,2,3,4,6)P5
Inositol hexakisphosphates
InsP6
scyllo-InsP6
P =
PP =
[32P]-labeled inositol pentakisphosphates
Ins([32P)1,3,4,5,6)P5
Ins(1,[32P]3,4,5,6)P5
Diphosphoinositol phosphates
(inositol pyrophosphates)
5-PP-Ins(1,3,4,6)P4
5-PP-Ins(1,2,3,4)P4
1-PP-InsP5 (1-InsP7)
3-PP-InsP5 (3-InsP7)
4-PP-InsP5 (4-InsP7)
6-PP-InsP5 (6-InsP7)
5-PP-InsP5 (5-InsP7)
1,5-[PP]2-InsP4 (1,5-InsP8)

Figure S2. Calibration curve for detection of $InsP_6$

A, an acid hydrolysate of sodium phytate (Sigma P8810) reconstituted in water at a concentration of 0.03 g/mL was diluted serially and 20 μL aliquots of x10, x20, x40 and x100 dilutions were injected onto HPLC eluted with methanesulfonic acid. **B**, a calibration curve for detector response to injected $InsP_6$.

Fig. S2

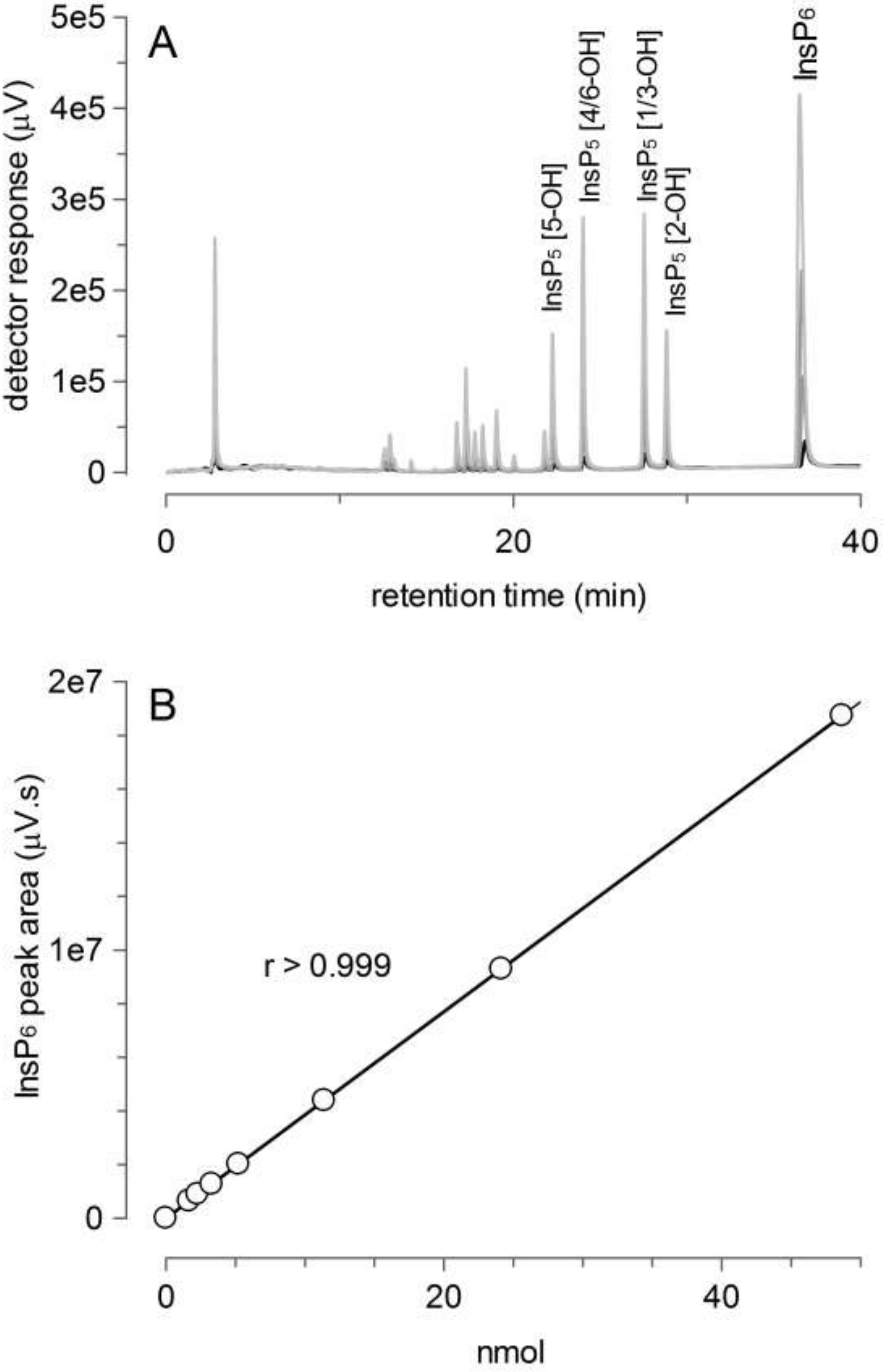

A
B
detector response (μV)
5e5
4e5
3e5
2e5
1e5
0
InsP5 [5-OH]
InsP5 [4/6-OH]
InsP5 [1/3-OH]
InsP5 [2-OH]
InsP6
retention time (min)
20
40
2e7
1e7
InsP6 peak area (μV.s)
r > 0.999
nmol

Figure S3. Arabidopsis ITPK1 hydroxykinase activity extends to $InsP_3$s. Consistent with phosphorylation of Ins(1,4,5,6)P_4 and Ins(3,4,5,6)P_4 on the 3- and 1- positions, respectively, ITPK1 shows phosphorylation of Ins(1,4,6)P_3 and Ins(3,4,6)P_3. Reaction products from incubations of ITPK1 with **A**, Ins(1,3,4)P_3; **B**, Ins(1,4,6)P_3 and **C**, Ins(3,4,6)P_3 are shown. Reactions were performed under non-ATP regenerating conditions. **D**, shows a set of standards obtained by acid hydrolysis of $InsP_6$. The HPLC column was eluted with a gradient of methanesulfonic acid. Reactions were performed in 20 mM HEPES pH 7.3, 6 mM $MgCl_2$, 10 mM $LiCl_2$, 1 mM DTT with 0.125 mM ATP, 1 mM $InsP_3$ and 10 µM enzyme. The structures of compounds described are shown in Figure S1.

Fig. S3

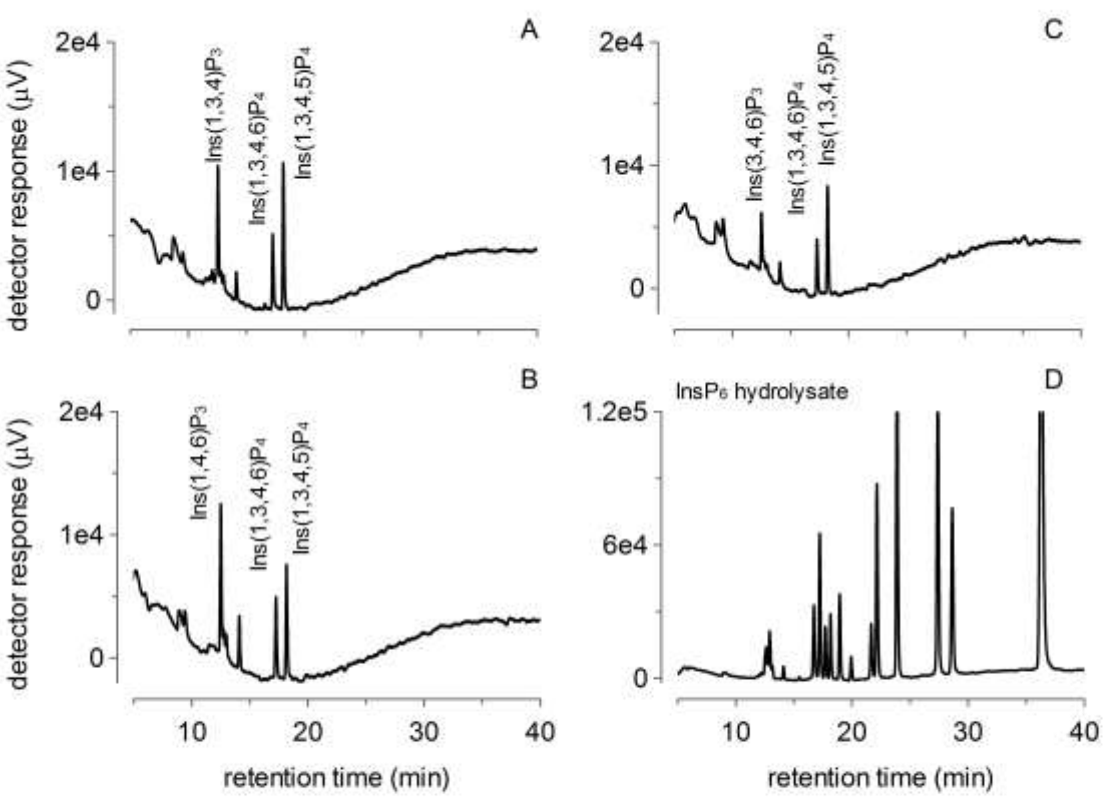

Figure S4. Sensitivity for detection of $InsP_6$ and 5-$InsP_7$ on HCl gradients. Products of reaction of ITPK1 with $InsP_6$ separated on an HCl gradient. In A, B and C equal amounts of total inositol phosphate were injected. The integrated peak areas (µV.s) are shown for $InsP_6$ and 5-$InsP_7$ peaks. In D, a peak of 5-$InsP_7$ representing 1.3 % of summed $InsP_6$ and 5-$InsP_7$ peak area is enlarged. Assays of 60 µL volume were performed in 20 mM HEPES, pH 7.5, 1 mM $MgCl_2$, 1 mM ATP, 1 mM $InsP_6$ in an ATP regenerating system with 3 µM enzyme. The structures of compounds described are shown in Figure S1.

Fig. S4

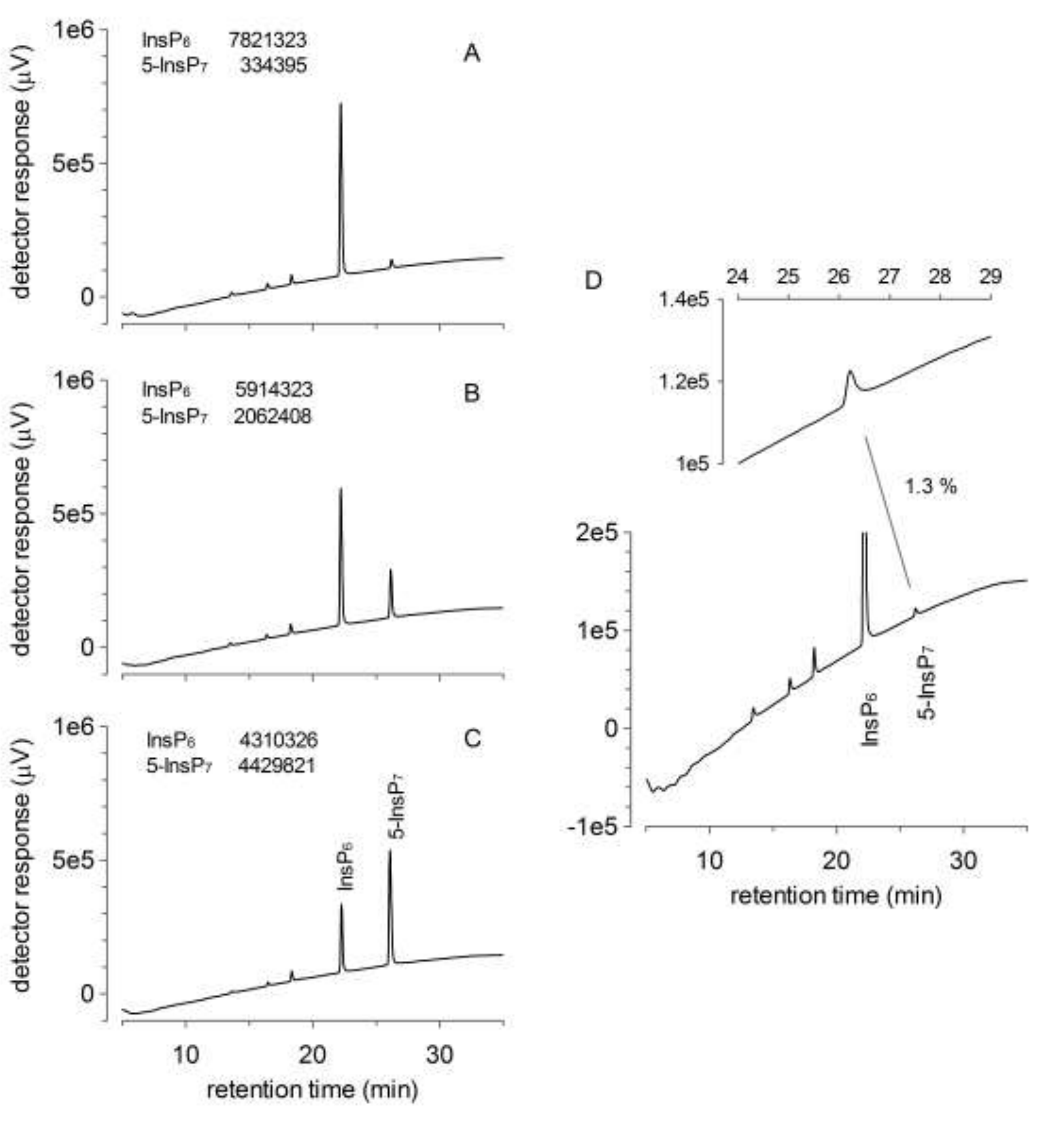

Figure S5. Inositol phosphate profiles of Arabidopsis seeds. Seeds extracted in cold 1M $HClO_4$, 5mM EDTA were analyzed on a CarboPacPA200 column eluted with an HCl gradient. A, C, or a methanesulfonic acid gradient, B, D. **A**, **B**, Col0, black line; *itpk1-1*, grey line; **C**, **D**, *mrp5*-2, black line; phytate hydrolysate, grey line.

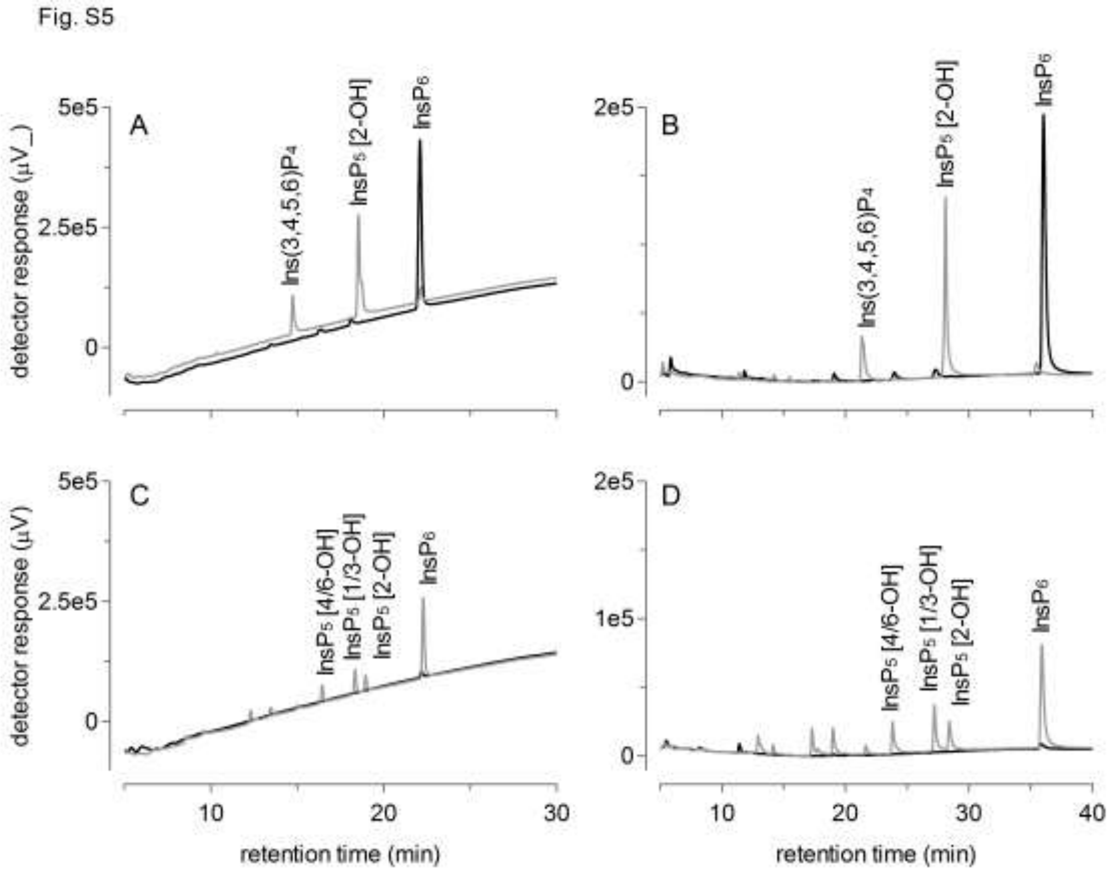

Figure S6. Detection of diphosphoinositol phosphates in rice bran and maize $InsP_6$ preparations. Preparations of $InsP_6$ from rice bran [1] and maize (Merck Millipore Product # 407125, maize dodecasodium salt) analyzed on an HCl gradient. **A**, approximately 200 nmol rice bran $InsP_6$, black line; with added *scyllo*-$InsP_6$, dark grey line; approximately 1 nmol *scyllo*-$InsP_6$, light grey; **B**, approximately 200 nmol rice bran $InsP_6$, same data set as A, black line; approximately 500 nmol maize $InsP_6$, thin black line; rice bran $InsP_6$ with added diphosphoinositol phosphates, dark grey line; approximately 0.1 nmol of each of 5PP-Ins(1,2,3,4)P_4, 5-$InsP_7$ and 3-$InsP_7$, light grey line. The diphosphoinositol phosphate region of the gradient is expanded in the insets (right), peaks of standards are identified. The different data sets were offset by different amounts on the *Y*-scale to aid visualization.

Fig. S6

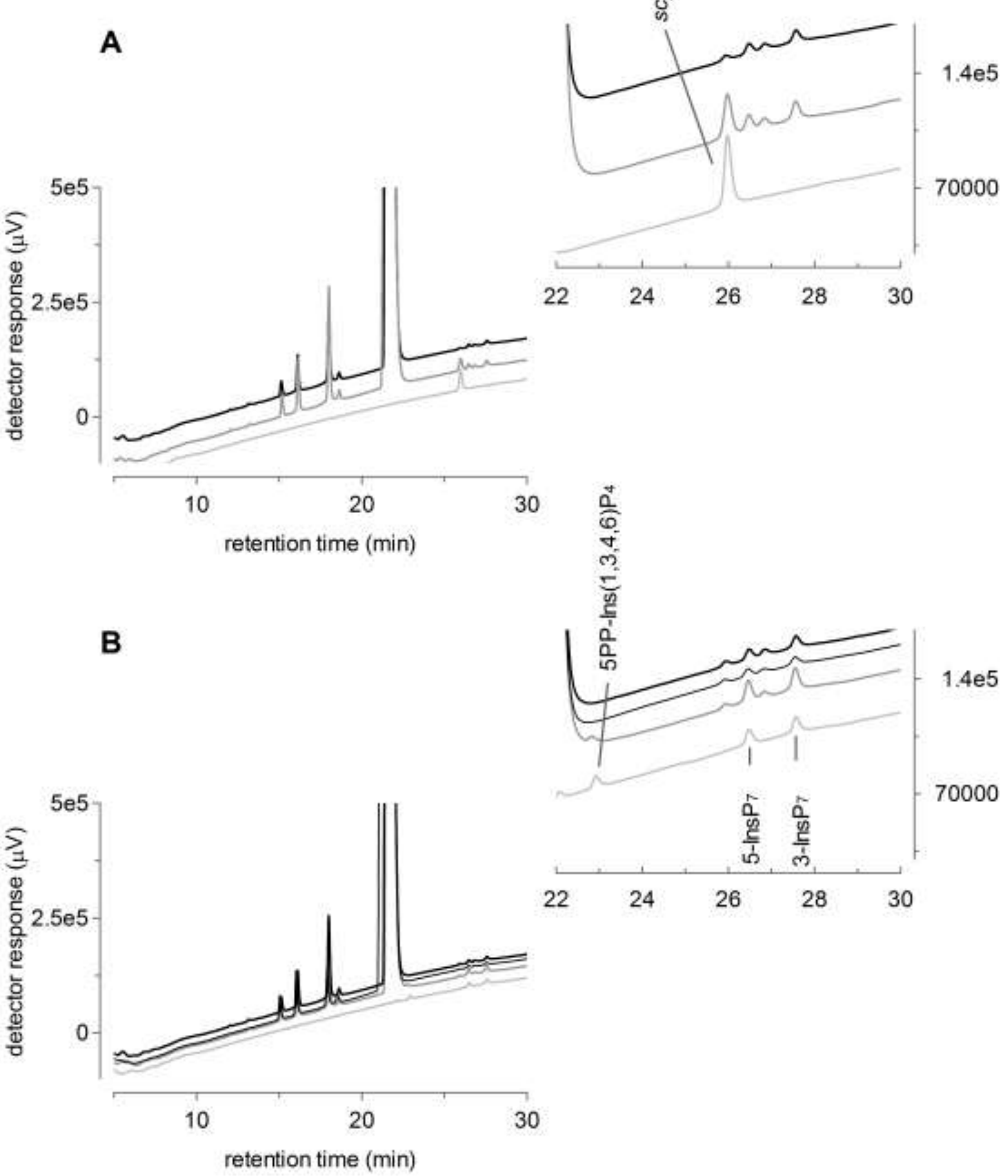
A
scyllo-InsP6
1.4e5
70000
22
24
26
28
30
5e5
2.5e5
0
detector response (µV)
10
20
30
retention time (min)
B
5PP-Ins(1,3,4,6)P4
1.4e5
70000
5-InsP7
3-InsP7
22
24
26
28
30
5e5
2.5e5
0
detector response (µV)
10
20
30
retention time (min)

Figure S7. Partisphere SAX HPLC separation of $InsP_5$s, putative 5PP-Ins(1,2,3,4)P_4, $InsP_6$ and 5PP-Ins(1,2,3,4,6)P_5 (5-$InsP_7$). Left *Y*-axis, cpm, black line; right *Y*-axis, gray trace, UV_{254}. Remnant nucleotides from phosphorylation reactions and acid-treatment of Ins([^{32}P]1,3,4,5,6)P_5 are labeled. $InsP_6$ was synthesized from Ins(1,3,4,5,6)P_5 and [γ-^{32}P] ATP with IPK1. Radiolabelled inositol phosphates were generated by mixing products of assays described in the legend to Figure 10. The structures of compounds described are shown in Figure S1.

Fig. S7

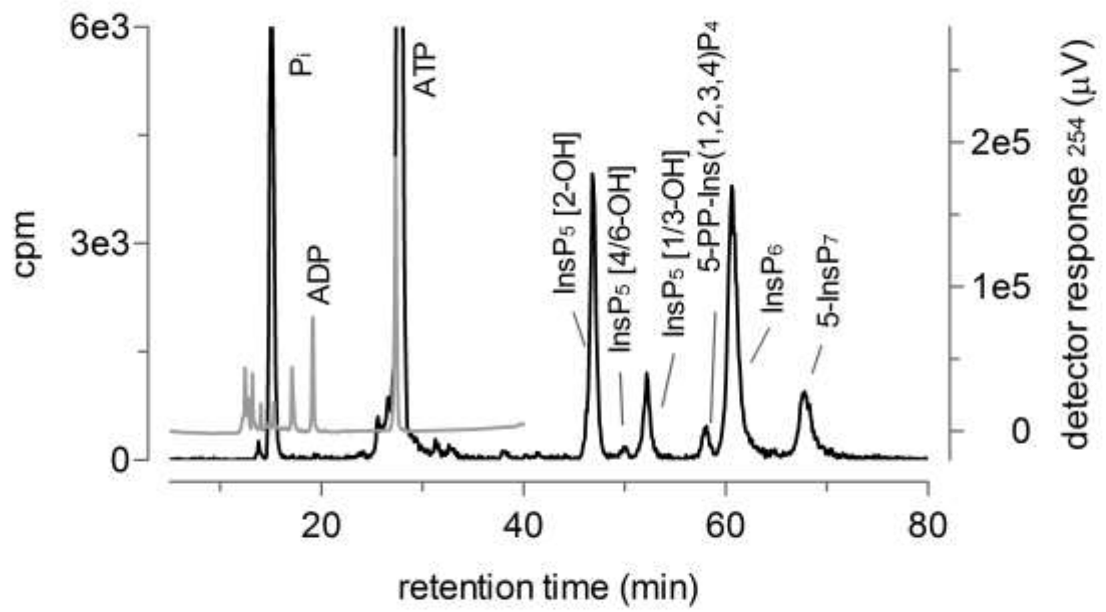

Table S1. Summary of Docking Results for Figures 4 and 7

Each substrate was docked against Human ITPK1 template. The poses were ranked by free energy (Rank 1 is the lowest energy pose). The lowest energy productive poses (that could explain the products formed) are indicated.

Substrate	Rank	Energy (kcal/mol)	Relative Energy (kcal/mol)[$]
Ins(1,4,6)P_3	2	-6.3	0.4
Ins(1,4,5,6)P_4	13	-6.1	0.5
Ins(3,4,6)P_3	3	-4.4	2.1
Ins(3,4,5,6)P4	4	-6.3	0.4
Ins(1,2,3,4,5)P_5	11	-0.9	5.7
InsP_6	10	5.0	8.7

[$] Relative to the energy minimum pose for each named substrate.

Table S2. Inositol phosphate hydroxykinase, phosphokinase and phosphotransferase reactions catalyzed by ITPK1 tested in this study

Substrate	Product	Preferred enantiomer	Reaction strength	Symmetry generating
Ins(1,3,4)P_3	Ins(1,3,4,6)P_4<br>D/L-Ins(1,3,4,5)P_4		*	*
Ins(1,4,6)P_3	Ins(1,3,4,6)P_4<br>D/L-Ins(1,3,4,5)P_4		*	*
Ins(3,4,6)P_3	Ins(1,3,4,6)P_4<br>D/L-Ins(1,3,4,5)P_4	*	*	*
Ins(1,2,4,6)P_4 †	-			
Ins(2,3,4,6)P_4 †	-			
Ins(1,3,4,5)P_4 †	-			
Ins(1,3,5,6)P_4 †	-			
Ins(1,3,4,6)P_4 †	-			
Ins(1,4,5,6)P_4 †	Ins(1,3,4,5,6)P_5		*	*
Ins(3,4,5,6)P_4 †	Ins(1,3,4,5,6)P_5	*	***	*
Ins(2,3,4,5,6)P_5 †	-			
Ins(1,2,4,5,6)P_5 †	-			
Ins(1,3,4,5,6)P_5 †	-			
Ins(1,2,3,5,6)P_5 †	-			
Ins(1,2,3,4,5)P_5 †	5-PP-Ins(1,2,3,4)P_4		*	
Ins(1,2,3,4,6)P_5 †	-			
Ins(1,3,4,5,6)P_5	Ins(3,4,5,6)P_4			
$InsP_6$ †	5-PP-Ins(1,2,3,4,6)P_5		*	*
5-PP-Ins(1,2,3,4,6)P_5	$InsP_6$			*
† ATP-regenerating conditions				
* Reaction strength within pair of enantiomers or class: e.g., among $InsP_4$s				
All compounds are named according to the 1D-numbering convention				

References

1. Madsen, C. K., Brearley, C. A. and Brinch-Pedersen, H. (2019) Lab-scale preparation and QC of phytase assay substrate from rice bran. Anal Biochem. **578**, 7-12
